# Evaluating the physicochemical effects of conjugating peptides into thermogelling hydrogels for regenerative biomaterials applications

**DOI:** 10.1093/rb/rbab073

**Published:** 2021-12-13

**Authors:** Hannah A Pearce, Emily Y Jiang, Joseph W R Swain, Adam M Navara, Jason L Guo, Yu Seon Kim, Andrew Woehr, Jeffrey D Hartgerink, Antonios G Mikos

**Affiliations:** 1 Department of Bioengineering, Rice University, 6500 Main Street, Houston, TX 77030, USA; 2 Depatment of Chemistry, Rice University, 6500 Main Street, Houston, TX 77030, USA

**Keywords:** thermogelling, click chemistry, hydrogels, peptides, poly(N-isopropylacrylamide)

## Abstract

Thermogelling hydrogels, such as poly(*N*-isopropylacrylamide) [P(NiPAAm)], provide tunable constructs leveraged in many regenerative biomaterial applications. Recently, our lab developed the crosslinker poly(glycolic acid)-poly(ethylene glycol)-poly(glycolic acid)-di(but-2-yne-1,4-dithiol), which crosslinks P(NiPAAm-co-glycidyl methacrylate) via thiol-epoxy reaction and can be functionalized with azide-terminated peptides via alkyne-azide click chemistry. This study’s aim was to evaluate the impact of peptides on the physicochemical properties of the hydrogels. The physicochemical properties of the hydrogels including the lower critical solution temperature, crosslinking times, swelling, degradation, peptide release and cytocompatibility were evaluated. The gels bearing peptides increased equilibrium swelling indicating hydrophilicity of the hydrogel components. Comparable sol fractions were found for all groups, indicating that inclusion of peptides does not impact crosslinking. Moreover, the inclusion of a matrix metalloproteinase-sensitive peptide allowed elucidation of whether release of peptides from the network was driven by hydrolysis or enzymatic cleavage. The hydrophilicity of the network determined by the swelling behavior was demonstrated to be the most important factor in dictating hydrogel behavior over time. This study demonstrates the importance of characterizing the impact of additives on the physicochemical properties of hydrogels. These characteristics are key in determining design considerations for future *in vitro* and *in vivo* studies for tissue regeneration.

## Introduction

Hydrogels that are injectable are a powerful tool in tissue regeneration that lend themselves to a variety of applications as they provide easy administration in a clinical setting and are space-filling [1–4]. Having been utilized in osteochondral tissue engineering [5–7], craniofacial tissue engineering [2] and myocardial tissue engineering [8], it is clear how versatile these injectable materials can be. Thermogelation of injectable materials is an advantageous strategy in tissue regeneration and drug delivery applications. This process minimizes the time the wound is open and also ensures homogenous distribution of additives suspended within the hydrogel, such as cells, microparticles, or drugs as the thermal gelation secures the components in 3D space rapidly. One of the most common ways in which thermogelation is achieved is through the use of poly(*N*-isopropylacrylamide) [P(NiPAAm)]. P(NiPAAm) is a polymer, which has been utilized extensively for a variety of thermogelling applications as it possesses a lower critical solution temperature (LCST) above which the network begins to gel [4, 9, 10]. This LCST can be modulated through the addition of varied co-monomers with more hydrophilic co-monomers increasing the LCST, and hydrophobic co-monomers lowering the LCST [4, 11]. This has been demonstrated through the co-polymerization of NiPAAm with glycidyl methacrylate to form P(NiPAAm-co-GMA), which reduces the peak LCST from 32 to 30°C, and even a four-part macromer with acrylic acid, glycidyl methacrylate and dimethyl-γ-butyrolactone acrylate (DBA). This four-part macromer possesses an initial LCST of 22°C and can be modulated to be as high as 60°C following hydrolysis of the lactone ring depending on the DBA content [1, 12]. This affords initial gelation at body temperature, but following incubation inside the body, the chains will solubilize as the lactone rings are hydrolyzed and will allow easier clearance from the body.

While efficacious, most thermogelling materials also employ chemical crosslinking. This creates a dual-gelling network in which thermal gelation space-fills a defect when injected into an environment above the LCST while chemical crosslinking secures and strengthens the network and prevents syneresis [1, 11, 13]. This dual-gelling strategy for injectable materials has proven to be useful in creating many robust tissue engineering materials capable of delivering cells, microparticles and bioactive factors.

Our group recently developed a novel crosslinking macromer, poly(glycolic acid)-poly(ethylene glycol)-poly(glycolic acid)-di(but-2-yne-1,4-dithiol) (PdBT) that crosslinks P(NiPAAm-co-GMA) through thiol-epoxy chemistry and permits degradation of the network via hydrolysis of the backbone ester bonds [9]. Moreover, PdBT can also be loaded with azide-bearing bioactive factors via azide-alkyne click chemistry to create a smart, bioactive hydrogel wherein the hydrogel undergoes gelation thermally at body temperature, rapidly crosslinks via thiol-epoxy reaction and presents bioactive factors within the network. We have demonstrated the ability to conjugate azide-modified N-cadherin mimic peptide HAV, osteogenic peptides GHK, BMHP1 and BMPm, as well as chondroitin sulfate [5, 9]. This presentation of bioactive factors made possible via PdBT has demonstrated the ability to promote chondrogenic and osteogenic tissue growth *in vitro* and *in vivo* [5, 6]. However, a mechanistic understanding of the impact of bioactive factor conjugation on the physicochemical properties of hydrogels is a gap in our understanding.

The impact of bioactive factor inclusion on hydrogel swelling and degradation are important characteristics to consider when designing a regenerative biomaterial system [9, 13]. Furthermore, the ability for the PdBT crosslinking macromer to present peptides within the network is important in long-term *in vitro* and *in vivo* applications. Many tissue regeneration solutions aim to offer bioactive factor presentation as wound healing, bone formation and mending and cartilage tissue engineering, among others, follow complex profiles of cell phenotypes and behavior that can be influenced by bioactive factor presentation [14–17]. In this work, the impact of peptides conjugated to PdBT on hydrogel physicochemical properties was investigated. Specifically, the impact of conjugating peptides within the network on the hydrogel sol fraction, swelling behavior, degradation, cytocompatibility and mechanism of peptide release were characterized. As thermogels and injectable materials are used widely in tissue regeneration, a mechanistic evaluation of how additives, such as peptides impact the material physicochemical properties is extremely valuable and can inform future *in vitro* and *in vivo* work for tissue regeneration.

## Experimental section

### Experimental design

**Figure 1.**
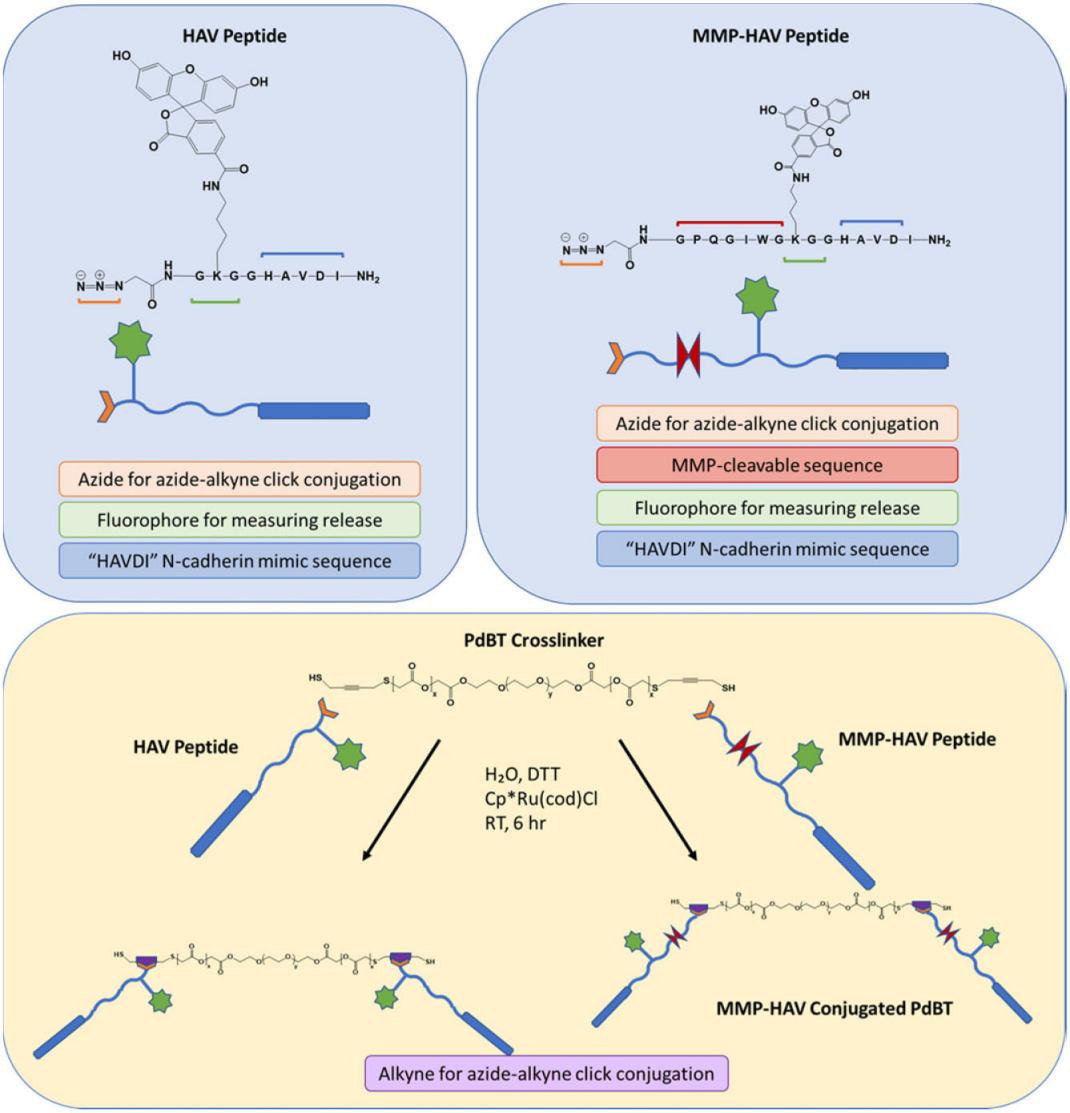
Schematic of the peptides designed for this study and peptide conjugation strategy. Both peptides were designed with an azide group for azide-alkyne click functionalization to the PdBT crosslinker that has an alkyne group on the backbone and a fluorophore for tracking release. The MMP-HAV peptide was designed with an MMP-sensitive sequence to study if release could be enzymatically controlled. Each peptide can be conjugated to the PdBT crosslinker via azide-alkyne click chemistry. The alkyne backbone pieces on the PdBT macromers are represented by the purple pentagons

Two model peptides were designed as shown in Fig.  1 to assess the impact of peptide conjugation within the hydrogel on the bulk hydrogel physicochemical properties.

N-cadherin mimic HAV peptide was chosen due to its widespread and well-characterized use as a pro-chondrogenic peptide utilized in many tissue regeneration applications [5, 18, 19]. Matrix metalloproteinase (MMP)-labile HAV peptide was chosen to allow study of the mechanism of release (i.e. hydrolytic or enzymatic) as degradation, swelling and release studies were to be carried out in conditions containing collagenase designed to mimic the wound-healing environment. Both peptides were labeled with a fluorophore for ease of monitoring retention and release. The peptides were then conjugated to the crosslinker PdBT to form PdBT/HAV and PdBT/MMP-sensitive N-cadherin mimic (MMP-HAV) and combined with PdBT in the mixtures established previously in our lab and reported below in Table  1.

**Table 1.** Hydrogel groups used in this study

	PdBT (mM)	PdBT/peptide conjugate (mM)	Dithiothreitol (DTT) (mM)
PdBT	25	0	100
PdBT/HAV	12.5	12.5	100
PdBT/MMP-HAV	12.5	12.5	100

Non-covalently loaded peptide controls were not included in this study given previous reports in the literature of rapid and burst release from hydrogels following hydrogel formation [20–22]. Preliminary studies were conducted in which the addition of reducing agents dithiothreietol (DTT) and tris(2-carboxyethyl)phosphine (TCEP) were added to PdBT in 4× molar excess prior to the crosslinker’s addition to the hydrogel precursor solution. As shown in Supplementary Fig. S1, the addition of DTT to PdBT and subsequent dual-crosslinking by DTT and PdBT within the gels resulted in equilibrium swelling behavior indicative of a well and homogeneously crosslinked network [4]. The equilibrium swelling of hydrogels crosslinked only with 25 mM PdBT was significantly less than its formation swelling. However, hydrogels crosslinked with 25 mM PdBT pretreated with 100 mM DTT possessed comparable formation swelling to the 25 mM PdBT group and greater equilibrium swelling than 25 mM PdBT + 100 mM TCEP, a reducing agent, which does not participate in crosslinking. These preliminary results demonstrated the combinatorial positive impact of utilizing DTT to both reduce PdBT prior to crosslinking, and also serve as a supplemental short-chain crosslinker to improve the crosslinking of the network. As such, a 4× molar excess of DTT was utilized to dually pre-treat all PdBT, PdBT/HAV and PdBT/MMP-HAV groups and serve as a short-chain supplemental crosslinker of the P(NiPAAm-co-GMA) hydrogels. Representative images of the hydrogels demonstrating a lack of thermoreversible gelation indicative of crosslinking can be found in Supplementary Fig. S2. Crosslinking times and LCSTs of the hydrogel mixtures were assessed as well as the sol fraction, swelling, degradation, peptide release and rheological properties of the hydrogels. Finally, the hydrogel leachables were assessed for cytocompatibility.

### Peptide synthesis

The azide-bearing N-cadherin mimic [Azide-‘GK(MTT)GGHAVDI’] (HAV) and MMP-HAV [Azide-‘GPQG*IWGK(MTT)GGHAVDI’] were synthesized as reported previously using solid phase synthesis and fluorenylmethoxycarbonyl-based chemistry using a rink amide MBHA resin [9, 23–26]. 5(6)-carboxyfluorescein was utilized to fluorescently label the peptides via selective de-protection of the methyltrityl group from the lysine residue prior to cleavage from the resin in a 2:2:96 mixture of trifluoroacetic acid:triisopropyl silane:dichloromethane for 1 h, followed by coupling with a 2–4× molar excess of the fluorophore for 2 h. Following peptide synthesis and cleavage from the resin, the product was collected via trituration with cold ether and dissolved in 10% acetic acid before dialyzing against MilliQ water for 24 h to purify as previously reported [5, 9, 27]. Matrix-assisted laser desorption/ionization (MALDI-TOF) mass spectrometry (Bruker AutoFlex Speed MALDI-TOF) was used to confirm synthesis of the correct peptide sequences using a 10 mg/ml peptide solution dissolved in 70:30 H_2_O:ACN containing 0.1% TFA and 1 µl of this solution was added to 9 µl of concentrated alpha-cyano-4-hydroxy-cinnamic acid (CHCA) matrix solution and spectra were collected scanning from 750–3500 Da.

### Hydrogel formation

PdBT and P(NiPAAm-co-GMA) were synthesized as previously described [4, 9, 13]. The structures of PdBT and P(NiPAAm-co-GMA) were assessed with ^1^H NMR using a 600 MHz Bruker NEO Digital NMR Spectrometer [4, 9, 13]. PdBT was additionally characterized via MALDI-TOF. The click conjugation of the azide-presenting peptides to PdBT was performed according to protocols previously established in the lab using a 2:1 molar feed of peptide to PdBT using the cytocompatible and water-soluble ruthenium catalyst chloro(pentamethylcyclopentadienyl)(cyclooctadiene)ruthenium(II) [5, 9]. The PdBT/peptide products were then dialyzed against MilliQ water to remove any residual initiator, or unreacted PdBT or peptides [5, 9, 24]. Mass spectrometry was performed using a Bruker AutoFlex Speed MALDI-TOF to evaluate the molecular weight of the PdBT/peptide conjugates. Briefly, the PdBT and PdBT/peptide products were dissolved in 70:30 H_2_O:ACN containing 0.1% TFA and 0.1% NaCl to assist with ionization at concentrations of 10 mg/ml and 1 µl of this solution was then added to 9 µl of concentrated CHCA matrix for MALDI-TOF analysis. Molecular weight ranges of 750–3250, 750–5000 and 750–6000 Da were scanned for PdBT, PdBT/HAV and PdBT/MMP-HAV, respectively [4, 13]. In addition to MALDI-TOF, PdBT, HAV, MMP-HAV and the PdBT/peptide products were analyzed via ^1^H NMR using a 600 MHz Bruker NEO Digital NMR Spectrometer.

**Figure 2.**
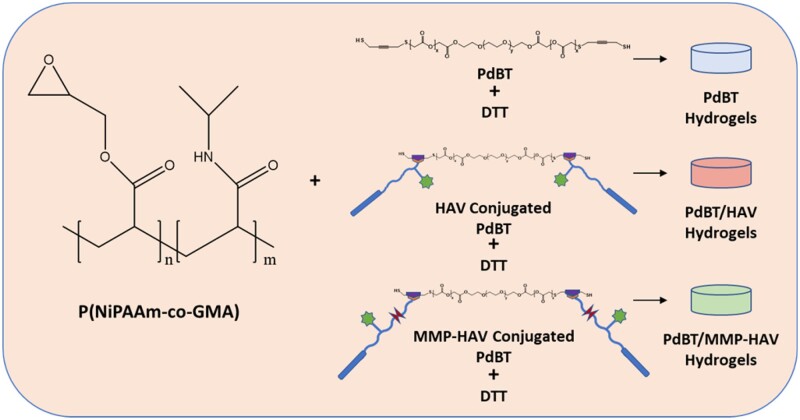
Schematic of hydrogel formation. P(NiPAAm-co-GMA) is crosslinked with PdBT, PdBT/HAV or PdBT/MMP-HAV and DTT as indicated in Table  1

The hydrogels for all studies were fabricated as previously described to form 10 w/v% P(NiPAAm-co-GMA) gels as depicted in Fig.  2 [5, 9].

The final PdBT macromer concentration used in this study was 25 mM as previously established in the lab as being the minimum PdBT concentration required for network formation. The PdBT/peptide groups consisted of a mixture of 12.5 mM PdBT and 12.5 mM PdBT/peptide as 12.5 mM is the upper solubility limit of the PdBT/peptide conjugates [5, 18]. The PdBT/peptide conjugates were dissolved in phosphate-buffered saline (PBS) + 10% dimethyl sulfoxide to aid in solubility, and PdBT was dissolved in PBS. DTT dissolved in PBS was added to the PdBT and PdBT/peptide crosslinker solutions in a 4× molar excess to reduce disulfides prior to gel formation and to serve as a supplemental short-chain crosslinker. The final working concentrations of the DTT, PdBT and PdBT/peptide conjugates were mixed according to the concentrations outlined in Table  1 with P(NiPAAm-co-GMA) to create a 10% w/v P(NiPAAm-co-GMA) solution. Gel precursor solution (30 µl) was pipetted into each well of a teflon mold (6 mm diameter × 1 mm height). The hydrogels were crosslinked for 24 h at 37°C in a controlled warm room. Prior to all analyses and following 24 h crosslinking, the sol fraction of the gels was removed by incubating the hydrogels in 4 ml PBS + 1% antibiotic-antimycotic at 37°C for 24 h [3, 28, 29]. The mass sol fraction (%) was calculated as the difference between the dried weight of the gel after 24 h crosslinking (*W*_d_) and the dry weight after 24 h incubation to remove the sol fraction (*W*_rd_) over the initial dry weight [(*W*_d_ − *W*_rd_)/*W*_d_ × 100] [28].

### Swelling and degradation

Following hydrogel formation, the swelling and degradation behavior were assessed as previously described [1, 4, 9, 11, 14, 27, 29, 30]. After crosslinking for 24 h at 37°C to form the hydrogels, the hydrogels were transferred and weighed in a pre-weighed 20 ml scintillation vial to obtain the formation weight (*W*_f_). The hydrogels were then swelled in PBS (pH 7.4) supplemented with 1% antibiotic-antimycotic for 24 h at 37°C and agitated at 100 rpm to remove the sol fraction. The hydrogels were then weighed in a scintillation vial to obtain the equilibrium swelling weight (*W*_sw, eq_). The hydrogels were frozen at −20°C overnight and lyophilized prior to measuring the initial dry weight (*W*_d0_). The initial formation and equilibrium swelling ratios were calculated as (*W*_f_ − *W*_d0_)/*W*_d0_ and (*W*_sw, eq_ − *W*_d0_)/*W*_d0_, respectively.

After removal of the sol fraction and measuring the initial dry weight, hydrogels (*n* = 3) were incubated in either PBS (4 ml) with 1% antibiotic-antimycotic (*n* = 3), or PBS containing 1% antibiotic-antimycotic, 800 ng/ml Type 1 collagenase [31] (Sigma Aldrich), and 150 nM CaCl_2_ at 37°C while agitated on a shaker plate at 100 rpm. This concentration of collagenase has been previously established as being representative of the wound-healing environment [31]. Swelling and degradation were evaluated on terminal timepoints (Days 1, 3, 7, 14 and 21) as previously described with media changes performed every 3 days [1, 9, 11, 14, 30]. At each terminal time point, the swollen weights were measured, and the sacrificial hydrogels were frozen and lyophilized. These hydrogels were subsequently washed with 10 ml MilliQ water overnight at 37°C while agitated at 100 rpm to remove salts before freeze-drying to obtain the dry weight at each time point (*W*_d_). The swelling ratio was calculated as (*W*_sw_−*W*_d_)/*W*_d_ at each time point. The percent mass loss at each time point was calculated as (*W*_d0_−*W*_d_)/*W*_d0_×100%.

### Peptide release and peptide sol fraction

As stated above, the sol fraction of the hydrogels was removed following 24 h crosslinking and prior to all analyses [3, 28, 29]. To assess the PdBT/peptide content in the sol fraction (*P*_s_), the fluorescence of the sol fraction for the PdBT/HAV and PdBT/MMP-HAV gels was compared against the fluorescence of known concentrations of PdBT/HAV and PdBT/MMP-HAV. To determine the amount of PdBT/peptide in the gel phase (*P*_g_), the gels were homogenized in PBS after collecting the sol fraction using a TissueLyser II (Qiagen) at a frequency of 240 Hz for 4 min and the fluorescence compared to known concentrations of PdBT/HAV or PdBT/MMP-HAV. All PdBT/peptide release was quantified as a percentage of the PdBT/peptide within the gel phase (*P*_g_). The PdBT/peptide content at hydrogel formation consisting of the sol and gel phases (*P*_f_) was then calculated as the sum of the PdBT/peptide in the sol phase (*P*_s_) and the PdBT/peptide in the gel phase (*P*_g_) [*P*_f_ = *P*_s_ + *P*_g_].

PdBT/peptide release was characterized following previously established methods [14, 30]. After removing the sol fraction, hydrogels from each group (*n* = 3) were freeze-dried and incubated in PBS + 1% antibiotic-antimycotic supplemented with 0 or 800 ng/ml collagenase type I. Release was carried out over the course of 21 days with medium changes performed every 3 days and was calculated as the cumulative PdBT/peptide released for the samples over the course of 21 days. All PdBT/peptide release was quantified by comparing the release supernatant against known concentrations of PdBT/peptide. [5] The supernatant obtained during each medium change and the peptide retained within the homogenized gels were assessed on a fluorescent plate reader (FLx800 or Cytation5, Bio-Tek Instruments) and compared to a standard curve of the respective PdBT/peptide conjugate at known concentrations [14, 30]. The excitation and emission wavelengths used to measure the PdBT/peptide conjugates were 485 and 528 nm, respectively.

MALDI-TOF mass spectrometry (Bruker AutoFlex Speed MALDI-TOF) was used to measure the molecular weight of degradation/release fractions of PdBT/HAV and PdBT/MMP-HAV hydrogels on Days 1, 3 and 6. Release medium (1 µl) was added to 9 µl of concentrated CHCA matrix solution prepared in 70:30 H_2_O:ACN containing 0.1% TFA. Spectra were collected scanning from 0 to 3500 Da for PdBT/HAV and 0–4500 for PdBT/MMP-HAV samples.

### Differential scanning calorimetry

The LCST and crosslinking times of the hydrogel mixtures were evaluated using differential scanning calorimetry (DSC) 250 (TA Instruments) as previously described [9, 11, 13]. P(NiPAAm-co-GMA) and the respective crosslinker were kept on ice before mixing immediately before each run. During each run, 14 µl of the gel solution was added to the sample pan. The sample was equilibrated to 15°C for 5 min, and then the temperature was ramped to 37°C and held constant for 300 min to assess the LCST and chemical crosslinking time [9]. The chemical crosslinking was considered complete when the heat exchange trended to 0 as described previously [9].

### Rheology

To assess injectability, rheology (Discovery HR1 hybrid rheometer, TA Instruments) was used along with 20 mm Peltier parallel steel plates to assess the gel point for each hydrogel group [11]. P(NiPAAm-co-GMA) and the respective crosslinker were kept on ice and only mixed immediately before injection onto the plate. The mixtures were equilibrated to 4°C for 5 min before ramping the temperature to 37°C at 10°C/min and held at 37°C until the end of the test. The mixtures were tested at a frequency of 1 Hz and strain of 1% until the solutions gelled and detached from the plates [1, 13]. The gel point was defined as the point at which the storage modulus (G’) crossed the loss modulus (G’’) [11].

### Leachables cytocompatibility

To assess the cytocompatibility of the hydrogel leachables, a leachable cytocompatibility assessment was performed as previously described [1, 4, 9, 11, 13, 27]. Briefly, passage 3 L929 fibroblasts were cultured in a T75 flask in L929 culture medium, which consisted of low glucose Dulbecco’s Modified Eagle Medium (LG-DMEM) (Gibco), 10% fetal bovine serum (Gibco) and 1% antibiotic-antimycotic until confluency at 37°C. The culture medium was changed every 3 days. After reaching confluency, the fibroblasts were replated in a 96 well plate at 10 000 cells/well and cultured in L929 culture medium for 72 h [1]. Hydrogel (*n* = 4) leachables were obtained by placing the hydrogels into serum-free LG-DMEM at a 1:1 surface area (cm^2^) to volume (ml) ratio immediately after crosslinking for 24 h at 37°C. The leachables medium for each hydrogel composition was then pooled, sterile filtered and diluted to 1×, 10× and 100× before placing 100 µl aliquots of each of the dilutions to expose the fibroblasts to the leachables (*n* = 4). Fibroblasts that were exposed to serum-free media were used as the control. The fibroblasts were incubated at 37°C for 2 and 24 h before evaluating the metabolic activity of the fibroblasts using a WST1 assay (MilliporeSigma). Their metabolic activity was normalized by DNA content assessed using a PicoGreen assay (ThermoFisher Scientific) [27].

### Statistical analysis

Data were analyzed using one-way (two groups) or two-way (three or more groups) analysis of variance followed by Tukey’s Honest Significant Difference *post-hoc* test (*P* < 0.05), Welch’s correction for varied standard deviation between groups, or Sidak’s multiple comparisons test when comparing different numbers of replicates (*P* < 0.05). All statistical tests were conducted and obtained using JMP and GraphPad Prism (GraphPad Software, La Jolla, CA). Data points are displayed as means ± standard deviation, unless otherwise noted.

## Results and discussion

Developing injectable hydrogels for tissue regeneration is of great interest given the utility of injectable systems and their ease of translation in a clinical setting [2, 6, 32]. Materials that possess the ability to dual-gel through both physical and chemical gelation are particularly powerful as thermal gelation can be leveraged to space-fill and set the material inside a defect, while chemical crosslinking can be employed to lock the network in place to prevent syneresis. P(NiPAAm)-based gels are an appealing option for thermogelling applications given the ability to easily tune the LCST to create one that is physiologically relevant [1, 13]. Additionally, the incorporation of drugs and bioactive factors is particularly attractive in hydrogels as these can be used to dictate cell behavior and guide tissue regeneration [2, 33, 34]. Many schemata have been employed to deliver bioactive factors, including peptides, to direct cell behavior and encourage tissue regrowth but little is known on how the inclusion of these factors impacts the physicochemical properties of their carrier hydrogels [5–7, 30]. One of the most influential contributors to the field of thermogelling materials is Dr Nicholas Peppas. His work characterizing hydrogels including P(NiPAAm) have impacted this study [10, 35–39]. In this work, the impact of the inclusion of peptides within the network on the physicochemical properties of a model P(NiPAAm-co-GMA) thermogelling hydrogel was characterized. Using the novel and recently developed PdBT crosslinker, N-cadherin mimic HAV peptide (HAV) and MMP-HAV peptide were conjugated to PdBT to form an injectable hydrogel network crosslinked with PdBT, PdBT/HAV and PdBT/MMP-HAV. Physicochemical characterization was performed to elucidate how the peptides influenced the swelling, sol fraction, degradation, LCST and rheological properties of the hydrogels. Finally, the mechanism of peptide release, whether hydrolytically driven or enzymatically driven, was investigated in an *in vitro* environment reflective of wound healing and inflammation.

### Hydrogel component fabrication and characterization

The PdBT crosslinker and P(NiPAAm-co-GMA) macromer were synthesized according to well-established methods and confirmed via ^1^H NMR [9]. The azide-bearing peptides HAV and MMP-HAV were synthesized, purified via dialysis as reported previously [5, 9, 27], and confirmed via MALDI-TOF mass spectrometry (Supplementary Figs S3 and S4) [40, 41]. Previous work has demonstrated the ability for peptides, such as HAV N-cadherin mimic peptide to maintain bioactivity following this azide-modification [5, 6]. From the mass spectrometry, the HAV peptide containing both the fluorophore and the azide was the only product detected. For the MMP-HAV peptide, the products were mixed (four in total). More details on the peptide products can be found in the Supplementary Material. Importantly, only the correct MMP-HAV sequence and the correct sequence minus the fluorophore could have reacted with PdBT as they were the only products containing an azide. While the MMP-HAV product missing the fluorophore would have still reacted with the PdBT, all PdBT/HAV and PdBT/MMP-HAV content within the sol and gel portions and all PdBT/peptide release was calculated from standard curves prepared from the PdBT/HAV and PdBT/MMP-HAV products. Thus, the PdBT/HAV and PdBT/MMP-HAV concentrations would still be calculated appropriately based on known concentrations of the PdBT/peptide products. The HAV and MMP-HAV peptides were combined with PdBT in a 2:1 peptide: PdBT molar ratio and following reaction and dialysis were confirmed using MALDI-TOF mass spectrometry. The ^1^ H NMR spectra for the peptides, PdBT, and their respective products can be seen in Supplementary Figs S5 and S6. The full MALDI-TOF spectra for PdBT, PdBT/HAV and PdBT/MMP-HAV can be found in Supplementary Figs S7–S10 [40, 41]. Zoomed in spectra for PdBT/HAV and PdBT/MMP-HAV as well as their chemical structures can be found in Supplementary Figs S9 and S11, respectively. From mass spectrometry analysis, conjugation of one peptide to each PdBT was demonstrated resulting in molecular weights of 2519 Da for PdBT/HAV and 3803 Da for PdBT/MMP-HAV. The molecular weight corresponding to the conjugation of two peptides per PdBT was not present in the mass spectrometry spectra for PdBT/HAV and PdBT/MMP-HAV. The presence of the fluorophore and the large molecular weights of the peptides (1292 Da for HAV and 1933 Da for MMP-HAV) likely limited the efficiency of the azide-alkyne conjugation due to steric hindrance and chain entanglement. This is in alignment with previous reports of azide-alkyne peptide conjugation to PdBT, which has resulted in ∼50–60% mass yield for the reaction [9]. Further, after dialysis to yield the purified product, NMR analysis demonstrated only ∼10% unreacted peptide remained in the mixture. This suggests that dialysis is capable of purifying the PdBT/peptide products so that they contain only small amounts of unreacted peptide [9]. As observed in the mass spectrometry data reported for this work, the PdBT/peptide products were mixed and peaks for unreacted PdBT and peptide are visible in the MALDI-TOF spectra. While dialysis as a method of purification is an inherent limitation, further purification was not possible due to solubility constraints. This has been reported for other large macromolecules in the literature as well [40, 41]. Given previous reports of dialysis removing all but 10% unreacted peptide following PdBT conjugation [9], it is reasonable to assume that PdBT/peptide were the primary products and thus the small amounts of unreacted PdBT or peptide would contribute minimally to the effects observed in this work.

To assess the impact of the crosslinkers on the LCST of the P(NiPAAm-co-GMA), the LCSTs of the hydrogel precursor solutions were evaluated via DSC. The LCST of the P(NiPAAm-co-GMA) was determined to be 30.3°C, which is similar to the LCST of 30.9°C previously reported in literature [13]. The PdBT macromer slightly lowered the LCST of the P(NiPAAm-co-GMA) to 29.5°C (Supplementary Fig. S12). Decreasing the LCST even more, the LCST of P(NiPAAm-co-GMA) mixed with PdBT/HAV and PdBT/MMP-HAV were 26.8 and 27.1°C, respectively. Importantly, the LCSTs were all between the desired range of room temperature, 25°C, and body temperature, 37°C, indicating their workability at room temperature prior to injection in the body to initiate gelation [32, 42].

**Figure 3.**
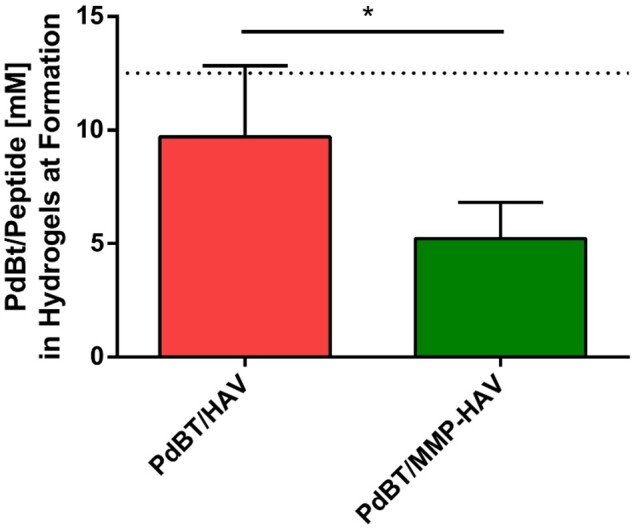
Maximum concentration of peptide contained within the gels after formation consisting of the sol and gel fractions. The dotted line at 12.5 mM indicates the concentration of PdBT/peptide conjugate added to the hydrogel precursor solutions. * indicates statistically significant difference (*n* = 4, *P* < 0.05)

The crosslinking times of the gels reacted with PdBT and PdBT/peptide were also assessed via DSC. The crosslinking time for the PdBT gels to reach completion was ∼60 min (Supplementary Fig. S13), which is comparable to the crosslinking time that has been previously reported for P(NiPAAm-co-GMA) crosslinked with PdBT [9]. The inclusion of the PdBT/HAV and PdBT/MMP-HAV crosslinkers increased the crosslinking time to ∼180 and ∼90 min, respectively. Interestingly, the PdBT/MMP-HAV crosslinker resulted in a shorter crosslinking time compared to the PdBT/HAV linker. This shorter crosslinking time may be attributed to the lower concentration of PdBT/MMP-HAV in the hydrogel at formation (sol and gel fractions) compared to PdBT/HAV (Fig.  3), leading to less interference in the P(NiPAAm-co-GMA) crosslinking with the PdBT and DTT in the mixture.

Previously, our lab has reported crosslinking with PdBT/HAV to be completed after 60 min [9], but the presence of the fluorophore on the PdBT/HAV and DTT in the mixture likely contributed to the slower crosslinking observed in this study. Previous reports have demonstrated that the addition of components, which raise the LCST (i.e. hydrophilic co-monomers) or large components, which physically impede P(NiPAAm-co-GMA) chain collapse can slow thermal gelation and allow for longer crosslinking times. This may explain what is occurring here for the PdBT/HAV group [4]. Nevertheless, all gels had finished reacting with the crosslinkers as analyzed via DSC within 3 h.

Rheology was utilized to elucidate the impact of incorporating peptides on the gelation of the hydrogels. The gel point was defined as the time at which the storage modulus (G’) was greater than the loss modulus (G’’) prior to the plateau [11]. The gel points for PdBT, PdBT/HAV and PdBT/MMP-HAV hydrogels occurred at 7.7, 5.7 and 7.5 min, respectively (Supplementary Fig. S14), with the first 5 min for all gels being a 4°C equilibration. All hydrogels quickly gelled within 3 min after a temperature increase simulating injection and their onset and peak LCSTs fell between 29 and 37°C, indicating that the addition of peptides do not impede the ability to be injected into the body and form a gel rapidly [32, 42]. As evidenced from the rheology, the LCSTs of the hydrogel mixtures appear to influence initial gelation. This thermodynamic process of P(NiPAAm) driving thermal gelation amidst the chemical crosslinking reaction has been documented previously [4]. As shown by the DSC data above, the hydrogel precursor solutions containing PdBT/HAV had the lowest LCST of 26.8°C and those with PdBT had the highest LCST of 29.5°C with the crosslinking times ranging from 60 min to 3 h. Taken together, the rheology data demonstrates that the thermal transition initiated by surpassing the LCST of P(NiPAAm-co-GMA) is sufficient to cause the mixture to gel even when crosslinking is not complete. In addition to the LCST, the molecular weight of the crosslinkers can influence gelation as molecular weight is known to impact reaction kinetics and viscosity [4, 43, 44]. This combinatorial effect of the molecular weights increasing viscosity and promoting more chain entanglement that helps drive thermal gelation, balanced with the difference in reaction kinetics between the P(NiPAAm-co-GMA) and the DTT, PdBT, PdBT/HAV and PdBT/MMP-HAV is expected to have impacted the rheological properties observed. Importantly, all hydrogel formulations were gelled within 3 min of ramping from 4 to 37°C. This demonstrates that the addition of peptides within a model P(NiPAAm-co-GMA) hydrogel did not negatively affect the gelation of the hydrogels [32, 42, 44].

### Sol fraction analysis

**Figure 4.**
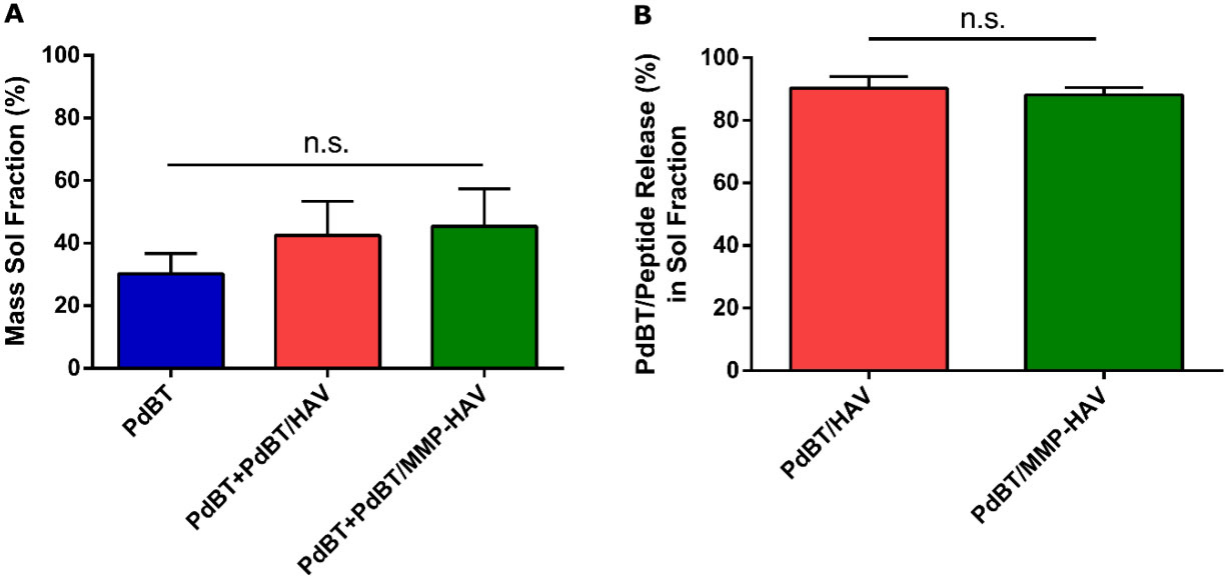
(**A**) Mass sol fraction of all hydrogel groups calculated following formation. No statistical significance was detected between groups (*n* = 4, *P* < 0.05). (**B**) Percent of PdBT/peptide conjugates released during the sol fraction. No statistical significance was found (n.s.) (*n* = 4, *P* < 0.05)

Following characterization of the rheological properties of the hydrogels and their dual thermal gelation and chemical crosslinking via DSC, their swelling behavior and sol fraction content were assessed. Immediately following crosslinking, the hydrogels were weighed (*W*_f_) and swelled in an excess of PBS for 24 h to remove the sol fraction [3, 14, 28, 29]. Representative images of the hydrogels following sol fraction extraction can be found in Supplementary Fig. S15. The equilibrium swollen weight (*W*_sw, eq_) was taken, and the supernatant saved to quantify both the mass sol fraction (%) for all groups shown in Fig.  4A, and the amount of PdBT/peptide content in the sol fraction of the PdBT/HAV and PdBT/MMP-HAV groups shown in Fig.  4B. The mass sol fraction (%) was found to be comparable for the PdBT, PdBT/HAV and PdBT/MMP-HAV groups at 30.2 ± 5.6, 42.5 ± 9.4 and 45.4 ± 10.4%, respectively, as shown in Fig.  4A.

The similarities in their mass sol fractions indicate that even with the presence of peptides in the mixture, the overall gel fraction between groups were comparable [3, 28, 29]. Interestingly, the amount of PdBT/HAV present in the sol and gel fraction of the hydrogels at formation was 9.7 ± 0.9 mM and for PdBT/MMP-HAV was only 5.2 ± 0.5 mM, as indicated in Fig.  3. Since mass sol fraction was the same for all groups and total crosslinker content for the PdBT/HAV and PdBT/MMP-HAV groups was composed of 12.5 mM PdBT, 12.5 mM PdBT/peptide and 100 mM DTT, the difference in PdBT/HAV and PdBT/MMP-HAV incorporation in their sol and gel fractions indicate that more PdBT and DTT was present compositionally in the sol and gel portions of the PdBT/MMP-HAV gels compared to the PdBT/HAV gels. This is supported by the DSC data for crosslinking time reported above as well. Due to their dual-gelling nature, P(NiPAAm-co-GMA) hydrogels are simultaneously thermally gelling and chemically reacting with the crosslinkers in the hydrogel. As shown in the DSC, the PdBT/HAV gels continued to chemically react far longer than the PdBT/MMP-HAV gels at 180 min vs 90 min, respectively. This indicates that the thermal gelation of the PdBT/HAV gels did not proceed at such a rate where crosslinkers were expelled quickly from the network. For the PdBT/MMP-HAV gels, DSC demonstrates that the chemical crosslinking was complete at 90 min. Taken with the peptide incorporation data in Fig.  3, this indicates that for the PdBT/MMP-HAV gels, more PdBT/MMP-HAV crosslinker was expelled from the gels in favor of PdBT and DTT during their thermal transition. This peptide loss is notable as peptide concentration can be reduced in future studies since the fractional content within the hydrogel is far lower than the initial 12.5 mM added to the hydrogel precursor solution. Lower peptide content within hydrogels has in fact been reported to be advantageous for specific applications as induction of encapsulated cells can be compromised if the peptide concentration is too high [18]. Surprisingly, the amount of PdBT/HAV and PdBT/MMP-HAV released in the sol fraction were comparable though at 90.3 ± 1.1% and 88.1 ± 0.7%, respectively, as shown in Fig.  4B. This indicates that while more PdBT/MMP-HAV was excluded during hydrogel formation in favor of PdBT and DTT, the ability for PdBT/HAV and PdBT/MMP-HAV to incorporate within the gel fraction were comparable once the thermal transition was complete [45]. Given the comparable mass sol fractions between all groups, it can be inferred that while the crosslinker composition (PdBT vs DTT vs PdBT/peptide) was different for the PdBT/HAV and PdBT/MMP-HAV groups, their crosslinking densities and gel fraction were similar. This understanding of the hydrogel components, which constitute the sol and gel portions of the network, as well as the crosslinking efficiency of hydrogel components are vital in designing regenerative biomaterial constructs. The gel fraction and chemical composition of the gel fraction influence hydrogel swelling, degradation, and release and can change over time [4, 45]. As such, these were the next characteristics explored.

### Formation and equilibrium swelling behavior

**Figure 5.**
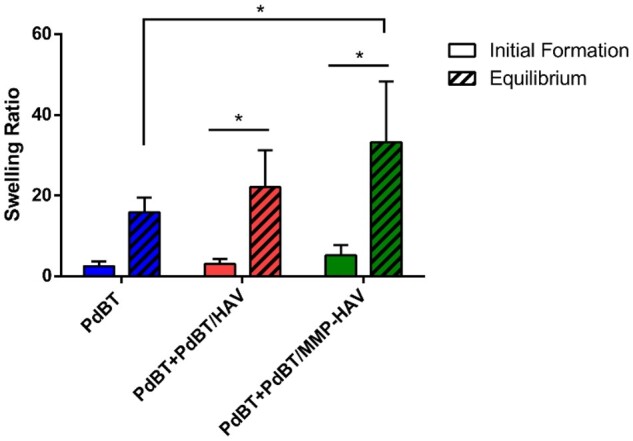
Formation and equilibrium swelling ratios of the hydrogels. * indicates statistically significant difference (*n* = 6, *P* < 0.05)

In comparing the formation swelling and equilibrium swelling of all groups, the equilibrium swelling ratio was greater than the initial formation swelling ratio for PdBT/HAV and PdBT/MMP-HAV hydrogels (Fig.  5), indicating the presence of a hydrophilic, perfusable hydrogel network (*P* < 0.05) [4].

Unlike other biomaterials, P(NiPAAm)-based hydrogels tend to swell more as they are chemically crosslinked since the polymer naturally will collapse and synerese on its own [1, 9, 11, 13]. The equilibrium swelling ratio of the PdBT/MMP-HAV gels was greater compared to the equilibrium swelling ratio of the PdBT gels (*P* < 0.05). This increase in equilibrium swelling compared to formation swelling has been reported previously when co-monomers or hydrogel constituents are hydrophilic, such as in the current case with the addition of HAV and MMP-HAV peptides [4, 46]. The findings outlined here for the PdBT/HAV and PdBT/MMP-HAV gels are consistent with these reports [4, 46, 47].

### Swelling and degradation

To assess the hydrogel degradation and swelling profiles over time, hydrogels were placed in PBS and PBS containing collagenase to mimic the wound-healing environment [27, 31]. Following removal of the sol fraction, hydrogels were dried, and their initial dry weight was recorded. The gels (*n* = 3) were then incubated in their respective media and removed at terminal timepoints (Days 1, 3, 7, 14 and 21). Each terminal timepoint consisted of sacrificial samples.

### Swelling behavior

**Figure 6.**
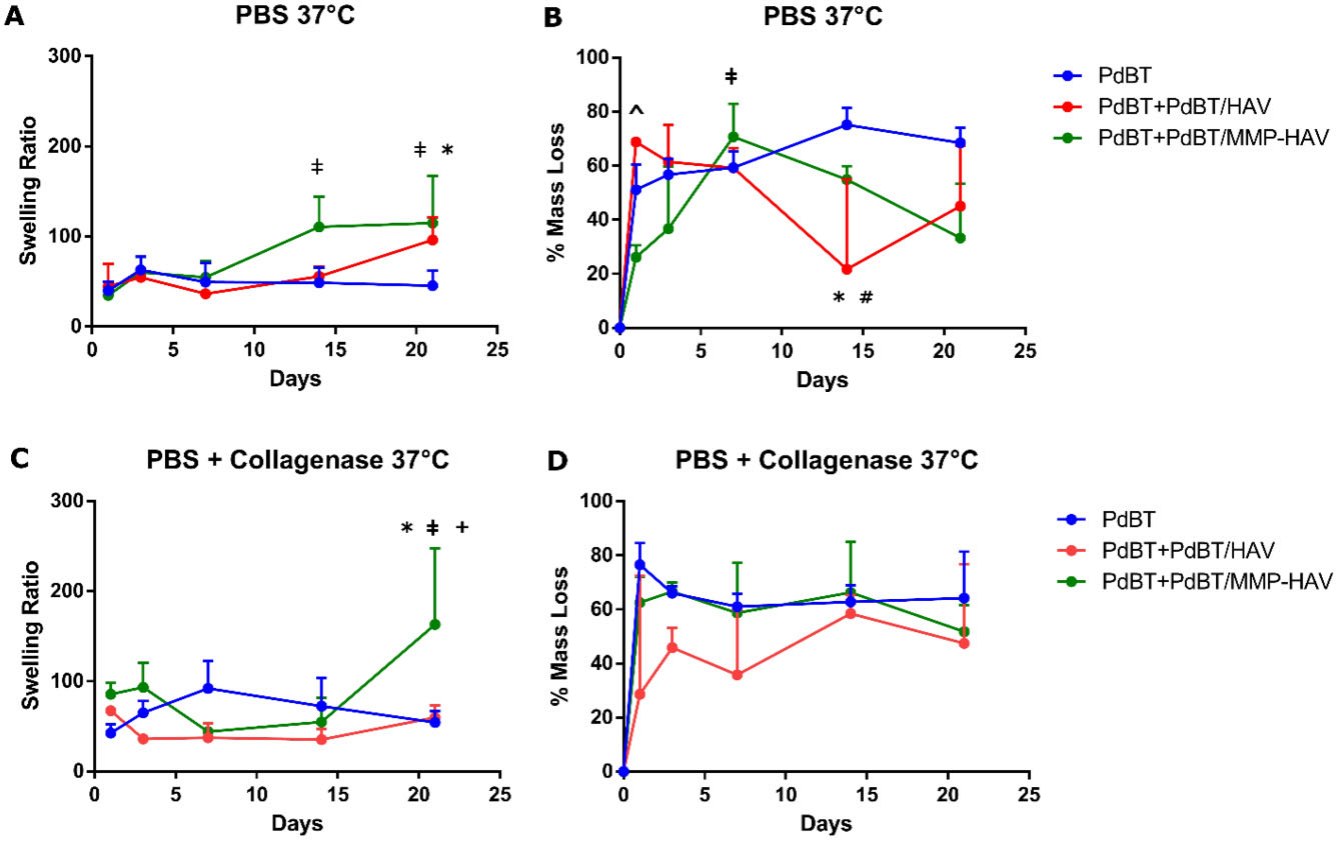
Swelling and degradation behavior of the hydrogels. (**A**) Swelling in PBS conditions. * indicates significance for PdBT/MMP-HAV group compared to PdBT control at Day 21 (*n* = 3–4, *P* < 0.05). ^**ǂ**^ indicates significance between PdBT/MMP-HAV Days 14 and 21 compared to Day 1. (**B**) Percent mass loss in PBS conditions. ^ indicates significance of PdBT/HAV compared to PdBT/MMP-HAV at Day 1. ^**ǂ**^ indicates significance between PdBT/MMP-HAV Day 7 and PdBT/MMP-HAV Day 1. * indicates significance between PdBT/HAV Day 14 and PdBT at that timepoint. ^**#**^ indicates significant difference between PdBT/HAV Day 14 and PdBT/HAV Days 1 and 3 (*n* = 3–4, *P* < 0.05). (**C**) Swelling in collagenase-containing conditions. * indicates significance of PdBT/MMP-HAV at Day 21 compared to PdBT at Day 21 and ^**+**^ indicates significance compared to PdBT/HAV at Day 21. ^**ǂ**^ indicates significance of PdBT/MMP-HAV Day 21 with respect to PdBT/MMP-HAV at Days 7 and 14. (**D**) Percent mass loss in collagenase containing conditions. After study initiation, no significant mass loss was observed between groups (*n* = 3–4, *P* < 0.05)

As demonstrated in Fig.  6A below, the swelling ratios of all hydrogel groups are similar in PBS conditions at Day 1 with means of 39.8 ± 8.8, 44.6 ± 20.4 and 34.5 ± 3.8 for PdBT, PdBT/HAV and PdBT/MMP-HAV, respectively.

The ratios remain similar to one another until Day 14 when the PdBT/MMP-HAV gels increase their swelling to 110.6 ± 27.6, which is significantly greater from the swelling for PdBT/MMP-HAV at Day 1 (^**ǂ**^, *P* < 0.05). By Day 21 the PdBT/MMP-HAV gels possess significantly greater swelling at 114.9 ± 42.8 than the PdBT controls at 45.5 ± 13.3 (*, *P* < 0.05) and the PdBT/MMP-HAV swelling at Day 1 (^**ǂ**^, *P* < 0.05). Throughout the course of the swelling study, the swelling for PdBT and PdBT/HAV groups are not significantly different from one another within each terminal timepoint. Additionally, the swelling for PdBT and PdBT/HAV and are not significantly different within their respective groups across days. This significantly increased swelling of PdBT/MMP-HAV compared to PdBT by Day 21 is consistent with the trends seen in the equilibrium swelling studies. Interestingly, there is no significant difference in swelling over time between PdBT/MMP-HAV gels and PdBT/HAV hydrogels within each timepoint. While PdBT/HAV swelling at Day 21 is not significant with respect to PdBT, PdBT/MMP-HAV and PdBT/HAV have comparable swelling by Day 21. The significantly greater swelling of the PdBT/MMP-HAV group compared to PdBT at equilibrium indicates an open, hydrophilic network, which continued to swell and expand over time [4]. Importantly, previous reports have established that hydrophilic, large molecular weight crosslinkers can serve as a barrier for P(NiPAAm) syneresis during crosslinking, which then results in a network composed of more crosslinks which can maintain integrity and resist syneresis and collapse [4]. The increased swelling of PdBT/MMP-HAV compared to PdBT at Day 21 is consistent with these findings and demonstrate the hydrophilicity of the network, which continues to promote swelling.

In collagenase-containing conditions shown in Fig.  6C, the peptide-bearing gels initially possess comparable swelling ratios to the PdBT controls. The comparable swelling among all three groups remains consistent for Days 3–14. By Day 21 again, PdBT/MMP-HAV possesses the highest swelling ratio at 163.2 ± 69.2 and is significantly greater than the PdBT control group at Day 21 with a ratio of 54.3 ± 10.2 (*, *P* < 0.05) and PdBT/HAV at Day 21 with a ratio of 59.8 ± 11.1 (^**+**^, *P* < 0.05). On day 21, the swelling of PdBT/MMP-HAV is also significantly greater than PdBT/MMP-HAV at Days 7 and 14 (^**ǂ**^, *P* < 0.05). When comparing PdBT and PdBT/HAV, the swelling does not significantly change within each group between days. This increased swelling for PdBT/MMP-HAV compared to PdBT by Day 21 supports what was observed in PBS conditions, though the significant increase with respect to PdBT/HAV is novel. Previous reports have indicated that P(NiPAAm)-based hydrogels can possess high water content when crosslinked with highly water absorbent co-monomers [4, 46]. Given this significant difference in swelling between PdBT/MMP-HAV and PdBT/HAV on Day 21 in collagenase-containing conditions, the enzyme’s action on the PdBT/MMP-HAV hydrogels appear to be significantly increasing their hydrophilicity compared to the enzyme’s effect on PdBT/HAV hydrogels. The continued expansion of the P(NiPAAm-co-GMA) network in the PdBT/MMP-HAV groups is promising for applications in future work given the tendency for P(NiPAAm-co-GMA) to synerese and collapse with prolonged degradation, preventing further release [3, 13].

### Degradation behavior

Assessing degradation in PBS, the PdBT/HAV gels initially lose significantly more mass by Day 1 than the PdBT/MMP-HAV gels ( ^, *P* < 0.05) as shown in Fig.  6B. From the swelling behavior at Day 1, no significant difference between groups is observed. As demonstrated in the release studies later, however, PdBT/HAV possesses high hydrolytic lability and thus resulted in significantly more degradation on Day 1 than PdBT/MMP-HAV. For PdBT/MMP-HAV in PBS, mass loss at Day 7 is significantly greater than that at Day 1 (^**ǂ**^, *P* < 0.05). The increase in mass loss for PdBT/MMP-HAV gels from Day 1 to Day 7 is intriguing when compared against swelling data. From the study, mass loss on Day 7 appears to precede the significantly increased swelling observed on Day 14. Interestingly, at Day 14, the mass loss in PBS for PdBT/HAV is significantly less than the PdBT group (*, *P* < 0.05) on Day 14 as shown in Fig.  6B. Mass loss for PdBT/HAV in PBS at Day 14 is also significantly less than PdBT/HAV mass loss at Days 1 and 3 (^**#**^, *P* < 0.05). The significant reduction in mass loss of the PdBT/HAV gels with respect to PdBT at Day 14 can be explained in part by the network collapsing and permitting less medium into the network for degradation. While the different crosslinkers may not have significantly impacted the swelling behavior between the PdBT and PdBT/HAV groups, the difference compositionally in their crosslinker content (PdBT vs PdBT+PdBT/HAV) can still impact degradation as degradation is driven by the network hydrophilicity and how accessible the hydrolytically labile bonds are [1, 9, 12]. On Days 14 and 21, the PdBT/MMP-HAV gels appear to lose less mass than on Day 7, similar to the trend observed for PdBT/HAV [3]. Nonetheless, the mass loss for PdBT/MMP-HAV at Day 7 is not significantly different from the mass loss at Day 14 or 21. For the Day 14–21 timepoints, degradation products could have adhered to the surface and acted to physically block degradation products from leaving the PdBT/HAV hydrogels resulting in their apparent weight gain. Furthermore, because each timepoint was terminal and consisted of sacrificial samples, sample-to-sample heterogeneity could explain in part the varied degradation profiles.

When assessing degradation in collagenase-containing conditions shown in Fig.  6D, no significant differences were observed between the three groups throughout the course of the study. Terminal mass losses for PdBT, PdBT/HAV and PdBT/MMP-HAV groups in collagenase-containing conditions were observed to be 64.2 ± 14.0, 47.5 ± 23.9and 51.8 ± 8.1%, respectively. The PdBT and PdBT/MMP-HAV degradation profiles are very similar, which could be an indicator of the MMP-HAV peptide being cleaved from the network and the gel thus behaving similarly to the PdBT control. Comparing mass loss to the swelling data for the collagenase-containing conditions, it is surprising that PdBT/MMP-HAV gels did not degrade more as they possessed the highest swelling ratio by Day 21. The increased swelling observed without degradation could correspond to increased hydrophilicity accomplished through hydrolysis of unreacted epoxy rings, cleavage of the esters in the PdBT crosslinker, or adsorption of the degradation products to the hydrogel surface [3].

### Peptide release

**Figure 7.**
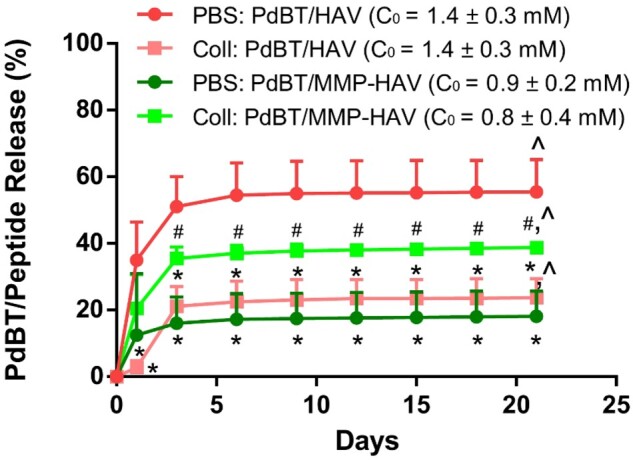
Release of the PdBT/peptide conjugates over time in PBS and PBS with collagenase. Initial concentrations (C_0_) are noted on the right-hand side demonstrating that initial dosage was not the only indicator for release. * indicates significant difference compared to PdBT/HAV in PBS at the same time point. ^#^ indicates significance compared to PdBT/MMP-HAV in PBS. ^ indicates significant release within a group from Day 1 to Day 21 (*n* = 3–6, *P* < 0.05)

To determine the mechanism of release of the peptides from the thermogels, N-cadherin mimic, HAV, and MMP-HAV peptides were designed and used as a model to study peptide release from the thermogels. Similar to all other studies, the hydrogels were crosslinked with PdBT, PdBT+PdBT/HAV or PdBT+PdBT/MMP-HAV and DTT as outlined in Table  1. Release from the network in PBS conditions was hypothesized to be based on initial PdBT/peptide content in the gel fraction and bulk degradation via hydrolysis. In collagenase-containing conditions though, it was hypothesized that the rate of release would be increased for the PdBT/MMP-HAV groups as release due to enzymatic activity would occur independently of bulk degradation via hydrolysis. As such, the concentration of PdBT/peptide in the gel fraction prior to release (*n* = 3 per group) was quantified so the impact of PdBT/peptide concentration, MMP-lability and degradation medium could all be examined in interpreting the results. To this end, the initial PdBT/peptide concentrations (C_0_) within the gel fraction for each group were calculated and are listed in Fig.  7. The PdBT/HAV hydrogels fabricated for release in PBS and PBS + collagenase contained similar PdBT/peptide gel fraction concentrations of 1.4 ± 0.3 mM for release in PBS and 1.4 ± 0.3 mM for PBS + collagenase. The PdBT/MMP-HAV gel fraction content within the groups fabricated for measuring release in PBS and PBS + collagenase was quantified to be 0.9 ± 0.2 mM for release in PBS and 0.8 ± 0.4 mM for release in PBS + collagenase.

First looking at PdBT/HAV, as shown in Fig.  7, the release of PdBT/HAV in PBS was found to be greatest with an ultimate release of 55.5%.

Interestingly, this is even greater than the release of PdBT/HAV in collagenase-containing conditions for all timepoints (*, *P* < 0.05), possessing a terminal release of only 23.7%. While PdBT/HAV does not possess MMP-labile bonds, one would expect that release of the PdBT/HAV in collagenase containing conditions to match the release of PdBT/HAV in PBS as both conditions permit release via hydrolysis. This indicates that while PdBT/HAV release was driven via hydrolysis, collagenase was masking the release of the PdBT/HAV from these hydrogels. A second explanation could be that collagenase was associating with the esters in the backbone of the PdBT/HAV and slowing the rate of hydrolysis. In comparing the degradation and swelling profiles of PdBT/HAV in PBS vs PBS + collagenase (data not shown), no significant differences were detected between groups within a timepoint indicating the medium did not significantly impact mass loss or swelling behavior of the PdBT/HAV hydrogels. This significant difference in release can be explained by collagenase associating electrostatically with the PdBT/HAV following hydrolytic degradation and hindering the release from the network, or blocking hydrolysis of the backbone esters, thus decreasing the apparent release measured here.

Looking at PdBT/MMP-HAV, release of PdBT/MMP-HAV in collagenase containing conditions is significantly higher than the release measured in PBS for Days 3–21 (^#^, *P* < 0.05) indicating that enzymatic cleavage, and not hydrolysis, is the primary driver for PdBT/MMP-HAV release from the hydrogels. The terminal release of PdBT/MMP-HAV in PBS conditions was only 18.2%. As seen for the PdBT/HAV release in collagenase containing conditions, collagenase could be associating with the esters of the PdBT/MMP-HAV backbone and reducing the rate of hydrolysis. Of note, PdBT/MMP-HAV release in collagenase-containing conditions is not significantly different than release of PdBT/HAV in PBS, though possessing a slightly lower release of 38.8%. This comparable release of PdBT/HAV in PBS and PdBT/MMP-HAV in PBS + collagenase is interesting as this means the rate of hydrolysis for PdBT/HAV matches the rate of enzymatic cleavage of PdBT/MMP-HAV from the hydrogels.

Comparing across linkers in PBS conditions, the release of PdBT/MMP-HAV in PBS conditions is significantly lower than release of PdBT/HAV in PBS at all timepoints (*, *P* < 0.05). This would indicate that compared to PdBT/HAV, hydrolytic release of PdBT/MMP-HAV was impeded. These data are interesting when taking into consideration the degradation data collected in PBS conditions. For the PdBT/HAV group, significantly more mass was lost by Day 1 than for the PdBT/MMP-HAV group as shown in Fig.  6B. This initial rapid degradation in PBS of the PdBT/HAV groups significantly impacted its peptide release, which was continued for the remainder of the release study. Surprisingly though, the increased swelling observed for PdBT/MMP-HAV in PBS observed on Days 14 and 21 and mass lost on Day 7 did not translate into increased PdBT/MMP-HAV release from the network. This retainment of the PdBT/MMP-HAV within the hydrogels in PBS conditions indicates that while the network was increasing in hydrophilicity over time, this did not result in hydrolytic release of the PdBT/MMP-HAV from the hydrogels.

MALDI-TOF mass spectrometry was used to measure the molecular weight of degradation/release fractions of PdBT/HAV and PdBT/MMP-HAV hydrogels on Days 1, 3 and 6 to aid in the interpretation of this release data. For PdBT/HAV hydrogels, the degradation fractions in PBS and PBS + collagenase appeared fairly similar, as seen in Supplementary Fig. S16. From the molecular weights, HAV and PdBT degradation fractions appear to be the majority of the products. For PdBT/MMP-HAV hydrogels however, the fractions look different in PBS vs PBS + collagenase as seen in Supplementary Fig. S17. For Days 1 and 3 in collagenase-containing conditions, a peak at 1500 Da is observed that is not seen in PBS conditions representing the sequence cleaved by collagenase (WGKGGHAVDI + Fluorophore). This peak is not observed in the Day 6 spectra, indicating that the majority of the MMP-labile peptide is released very quickly when relying on enzymatic-driven release. This supports what is observed in the release data where after Day 3, no significant changes in PdBT/MMP-HAV peptide release are detected.

The sequence of the MMP-cleaved peptide was calculated based on the molecular weight and did not include the anticipated isoleucine (I) as MMPs typically cleave between glycine (G) and isoleucine (I). Sequence specificity of the cleavage can be impacted by many things, such as the enzyme of choice and the neighboring amino acids in the sequence [25, 26]. For the peptide studied in this work, highly specific cleavage would have resulted in IWGKGGHAVDI + Fluorophore instead of the WGKGGHAVDI + Fluorophore observed. It is possible that cleavage was impeded given the proximity of the MMP-labile group to a fluorophore and the peptide being conjugated to the PdBT macromer. This can inform future work when considering the design of peptides for highly sequence specific action *in vitro* and *in vivo*. Considerations of peptide length and charge can also be investigated in future work to assess their roles in influencing physicochemical properties and release. Nevertheless, the release of the MMP-cleaved fraction observed via fluorescent measurement and by MALDI-TOF of the degradation products indicates successful cleavage of the MMP-HAV sequence from the network.

This systematic investigation of the mechanism of release of peptides from thermogels is essential as the degradation profile for hydrogels and the composition of their degradation products are important considerations for *in vitro* and *in vivo* work. Ultimately, all groups had released significantly more PdBT/peptide by Day 21 than at Day 1 ( ^, *P* < 0.05) aside from PdBT/MMP-HAV in PBS. This apparent resistance to hydrolytic release of the PdBT/MMP-HAV was surprising. This breaking from simple dose–dependent release via hydrolysis and a thorough investigation into the mechanism of peptide release in these two model hydrogels is vitally important when considering the broader impacts of using hydrogel carriers for tissue regeneration.

### Leachables cytocompatibility

**Figure 8.**
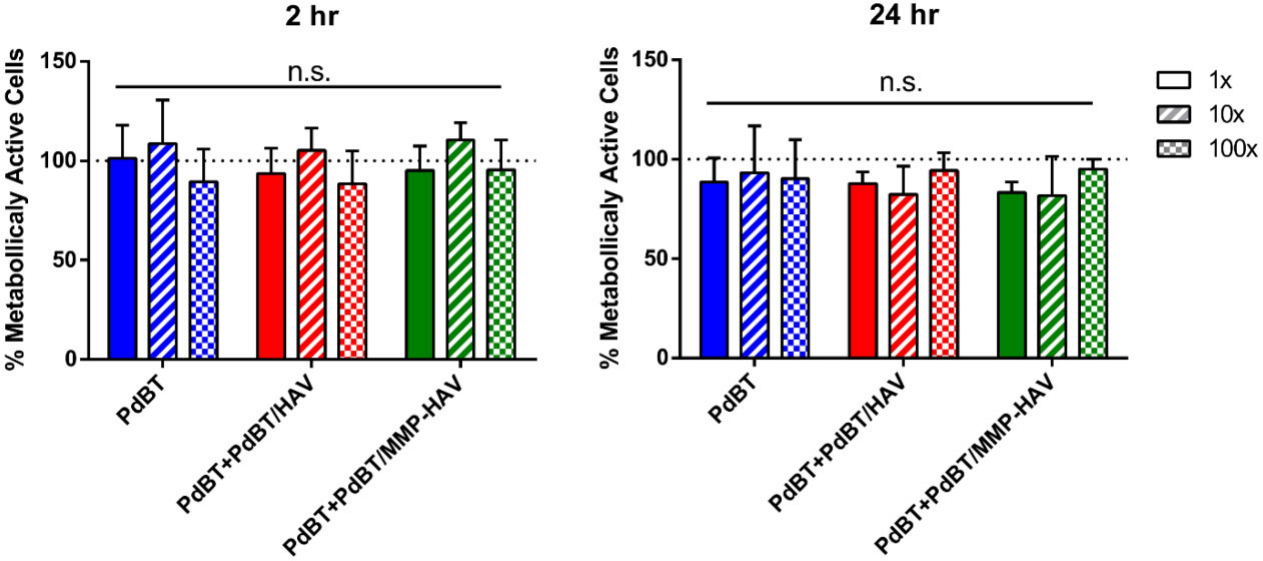
Metabolic activity as measured via WST1 assay normalized by DNA content measured by picogreen assay of fibroblasts exposed to hydrogel leachables for 2 and 24 h. No statistical significance (n.s.) was detected between groups within a timepoint (*n* = 4, *P* < 0.05)

Lastly, hydrogel leachables were evaluated for cytocompatibility for all groups as leachables cytocompatibility is an important indicator of future biocompatibility of hydrogel carriers for tissue regeneration [1, 9, 11]. L929 fibroblasts were exposed to leachables for 2 and 24 h at 1×, 10× and 100× dilutions. The metabolic activity of the fibroblasts normalized by the DNA content was not significantly different at the three different dilutions at 2 and 24 h for all groups (Fig.  8), demonstrating the cytocompatibility of the gel leachables even after peptide incorporation and that dialysis was sufficient for the purification of the PdBT/peptide as has been previously demonstrated [5, 9].

Moreover, the leachables experiments demonstrate that even with a sol fraction composed of unreacted crosslinkers (PdBT, PdBT/peptide and DTT), the leachables are still highly biocompatible. This demonstrates the potential for utilizing peptide incorporated thermogelling hydrogels as a cytocompatible carrier in future *in vitro* and *in vivo* applications.

## Conclusion

In conclusion, a systematic study was designed and performed to understand the impact of covalent peptide conjugation into a P(NiPAAm-co-GMA)-based thermogelling hydrogel. Two peptides were designed, an azide-presenting N-cadherin mimic HAV peptide and an azide-presenting MMP-HAV peptide, both labeled with a fluorophore. These peptides were covalently attached to a modular PdBT crosslinker via azide-alkyne click chemistry prior to their use as a crosslinker for the P(NiPAAm-co-GMA) hydrogels. The effect of the peptides within the network on the physicochemical properties of the hydrogels was assessed via sol fraction analysis, swelling, degradation and peptide presentation and release. Furthermore, the physical properties of the gels were evaluated via DSC to examine LCST and crosslinking time. Rheology was performed to assess the impact of the peptides on the system’s gel point to evaluate potential for injectability. The results reported here indicate the utility of P(NiPAAm-co-GMA) thermogelling hydrogels as a carrier for bioactive peptides and highlight important structure–property relationships when designing hydrogel constructs containing conjugated peptides. The addition of peptide-conjugated PdBT into the hydrogels did not influence mass sol fraction but increased equilibrium swelling for the PdBT/peptide groups compared to their formation swelling, indicating a crosslinked, hydrophilic network. Swelling and degradation of the PdBT/MMP-HAV group in collagenase containing conditions indicated that the network was becoming more hydrophilic without losing mass, which is an important consideration as hydrophilicity of thermogels raises the LCST and allows for easier clearance from the body. In examining PdBT/peptide release, it was determined that hydrolytic release of PdBT/HAV resulted in the most rapid release of the peptide conjugates, even outpacing the enzymatic cleavage of MMP-HAV. This work has demonstrated how peptide conjugation, peptide molecular weight and mechanism of peptide release can all influence the physicochemical properties of thermogels when covalently conjugated to the hydrogel network. Understanding these mechanistic pieces is invaluable and can be employed to aid in the design and interpretation of future tissue regeneration strategies *in vitro* and *in vivo*.

## Supplementary data

Supplementary data are available at *REGBIO* online.

## Supplementary Material

rbab073_Supplementary_Data
