# Supplementary material for: Evaluating the physicochemical effects of conjugating peptides into thermogelling hydrogels for regenerative biomaterials applications
Source: Regen Biomater. 2021 Dec 13;8(6):rbab073. doi: 10.1093/rb/rbab073 (PMC8684499; doi:10.1093/rb/rbab073)
Supplement: rbab073_Supplementary_Data [file rbab073_supplementary_data.zip › OP-REGB210073_PECorr_CmtAttachmentsFolder_Hydrogel Char Regen Biomater Supp Fig Resub 12.21.pdf.9.pdf]

Supplementary Figures

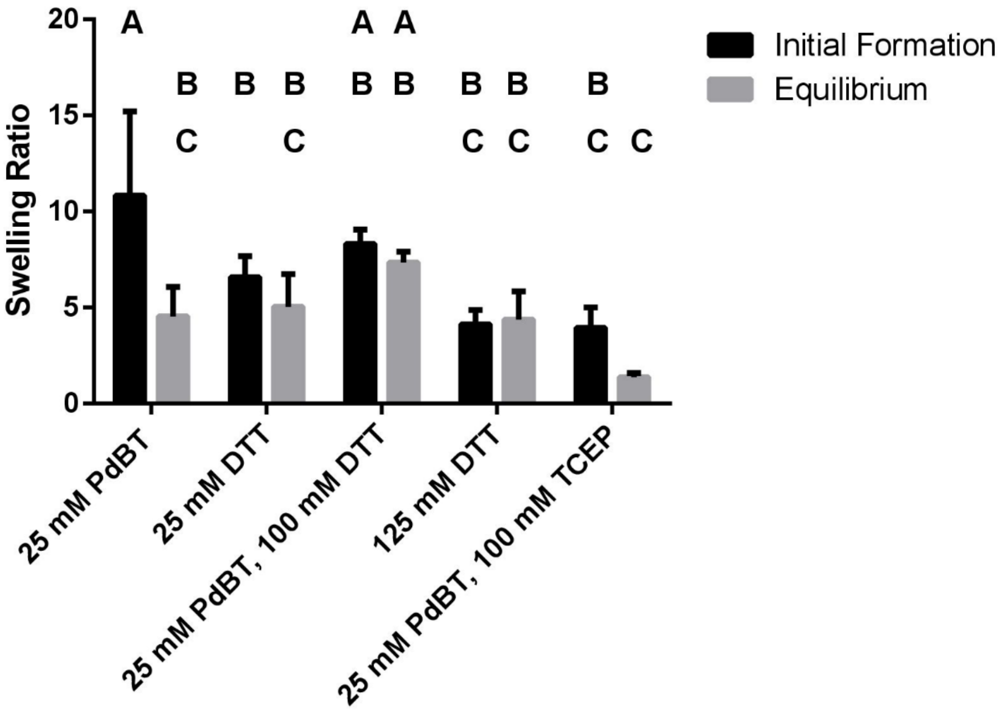

Supplementary Figure 1. Initial formation and equilibrium swelling data for preliminary gels demonstrating the combinatorial effects of PdBT and DTT in producing a highly robust and crosslinked network. Shared letters indicate lack of statistical significance (n = 4-6 per group, $p < 0.05$).

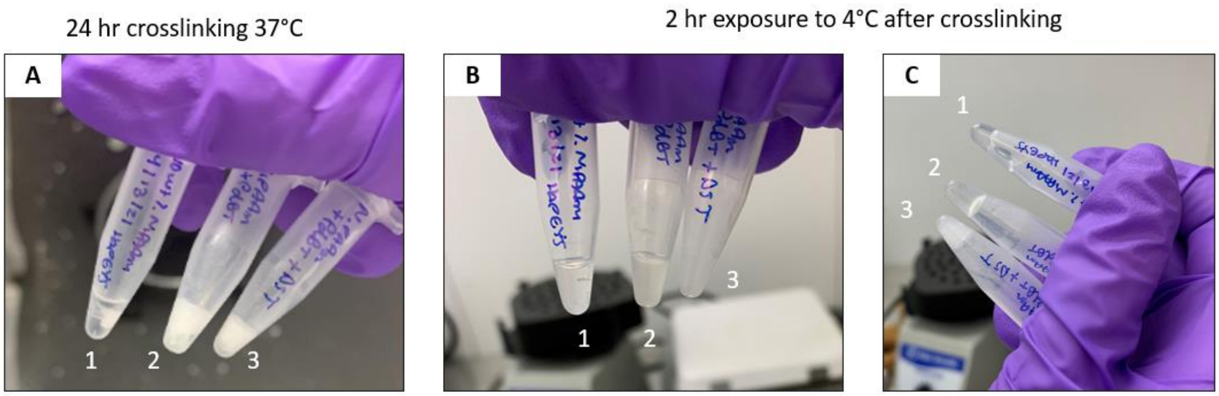

Supplementary Figure 2. Representative images of the hydrogels after crosslinking to assess if thermogelation is reversible by lowering the temperature below the LCST. Reversible gelation by lowering the temperature below the LCST indicates a non-crosslinked network. Tube numbers correspond to (1) P(NiPAAm-co-GMA) with no crosslinker, (2) P(NiPAAm-co-GMA) and 25 mM PdBT, and (3) (NiPAAm-co-GMA), 25 mM PdBT, and 100 mM DTT. The panels correspond to **(A)** hydrogels after 24 hr crosslinking at 37°C. **(B)** Hydrogels exposed to 4°C following 24 hr crosslinking and **(C)** the hydrogels inverted demonstrating reversal of the thermal gelation of product (1) following exposure to 4°C.

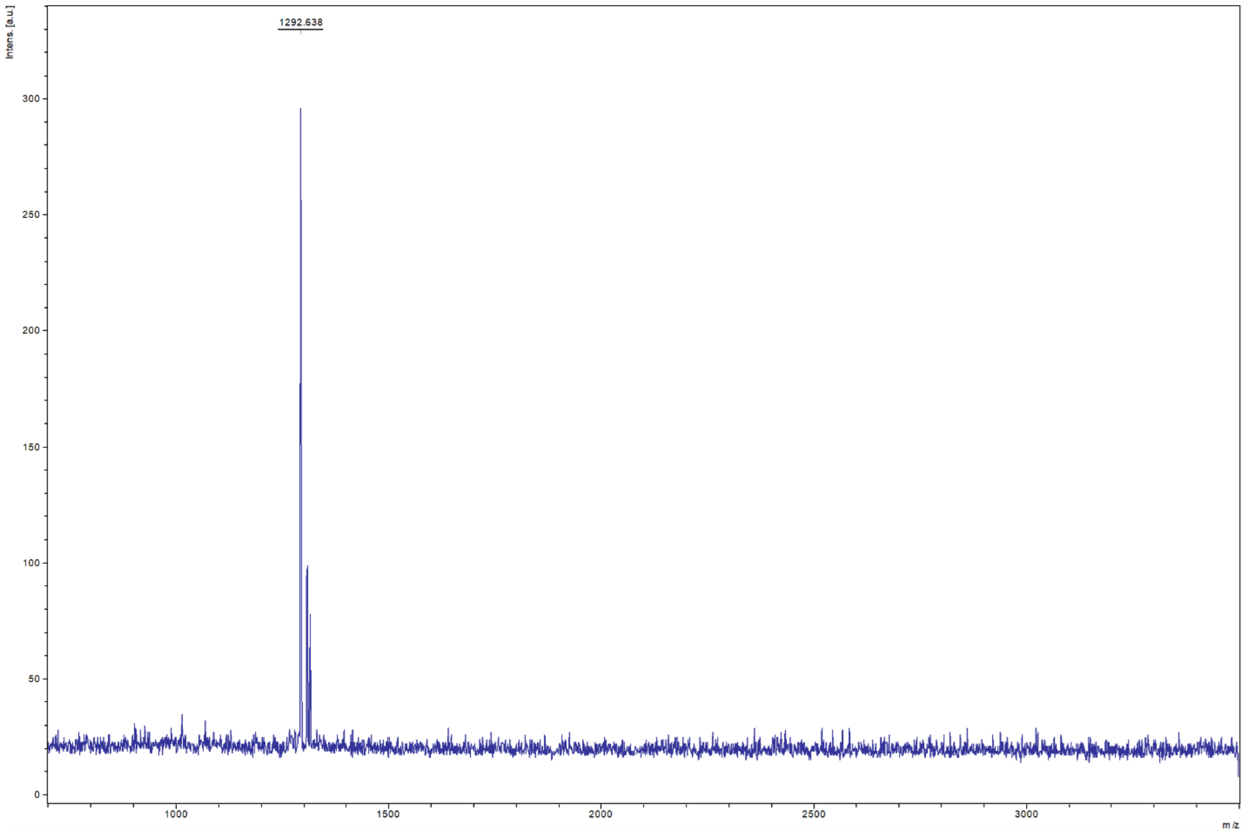

Supplementary Figure 3. MALDI-TOF spectrum of HAV peptide confirming structure. 1292 Da is the full peptide plus the azide and the fluorophore (Azide-“GK(Fluor)GGHAVDI” (HAV).

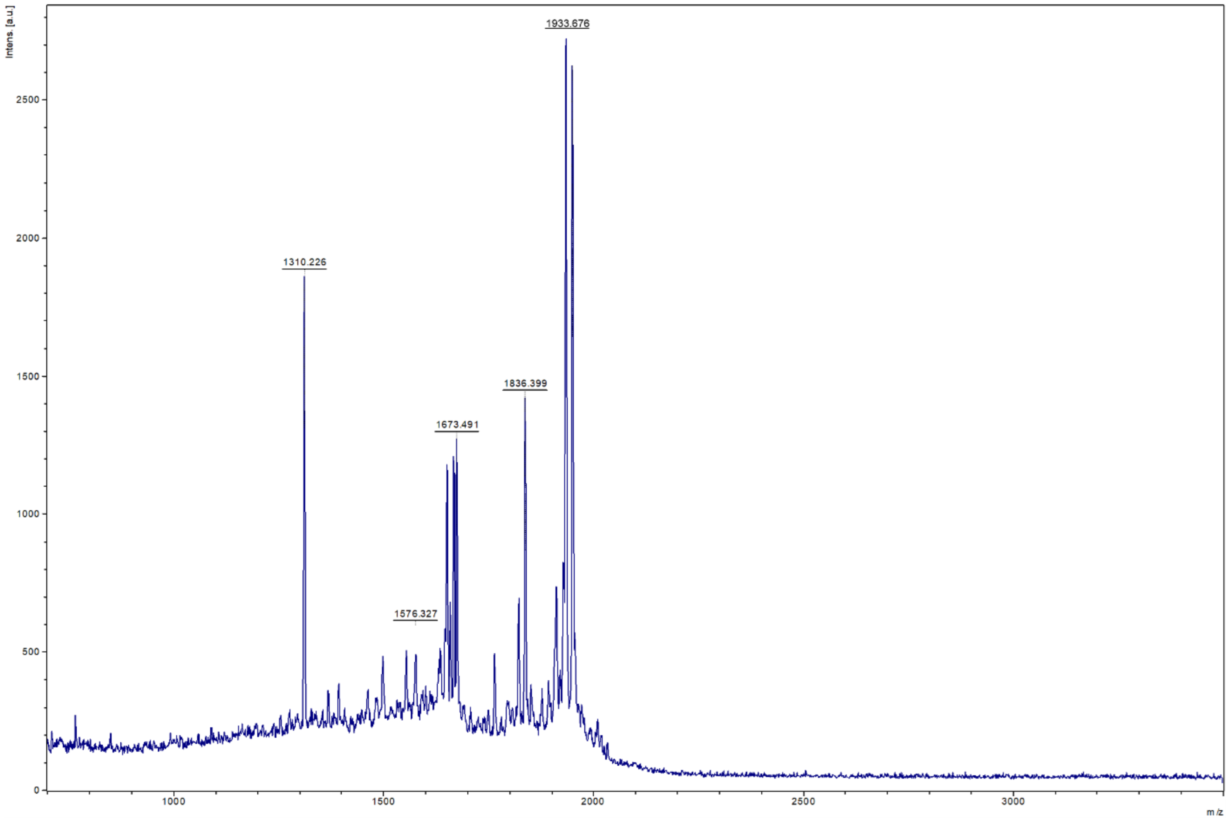

Supplementary Figure 4. MALDI-TOF spectrum of MMP-HAV peptide confirming structure. 1310 DA is the sequence “PQGIWGKGGHAVDI”. 1576 Da is Azide-“GPQGIWGKGGHAVDI” and 1673 DA is Azide-“GPQGIWGKGGHAVDI”+TFA, and 1836 Da is “GPQGIWGK(Fluor)GGHAVDI”. 1933 Da is the correct peptide sequence (Azide-“GPQGIWGK(Fluor)GGHAVDI”) (MMP-HAV).

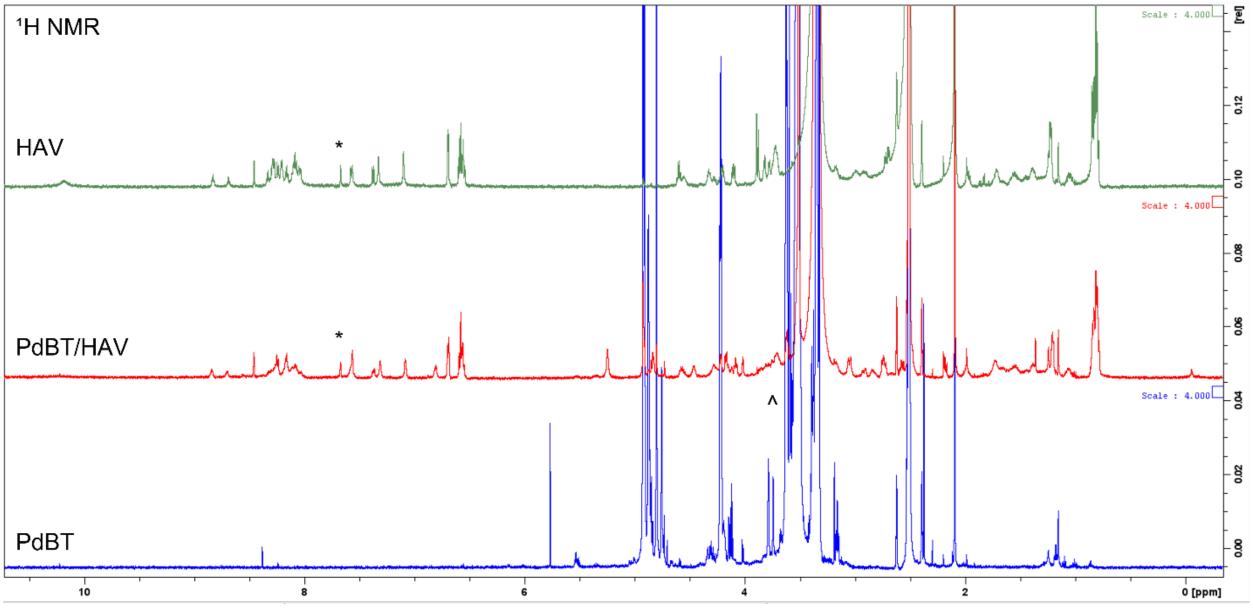

Supplementary Figure 5. ^{1}H NMR spectra of HAV, PdBT, and PdBT/HAV products dissolved in DMSO d6. The peak at 7.6 ppm (*) on the HAV and PdBT/HAV products correspond to the proton on histidine and the large peak at 3.54 ppm (^) on the PdBT and PdBT/HAV products correspond to the protons on the PEG backbone of PdBT.

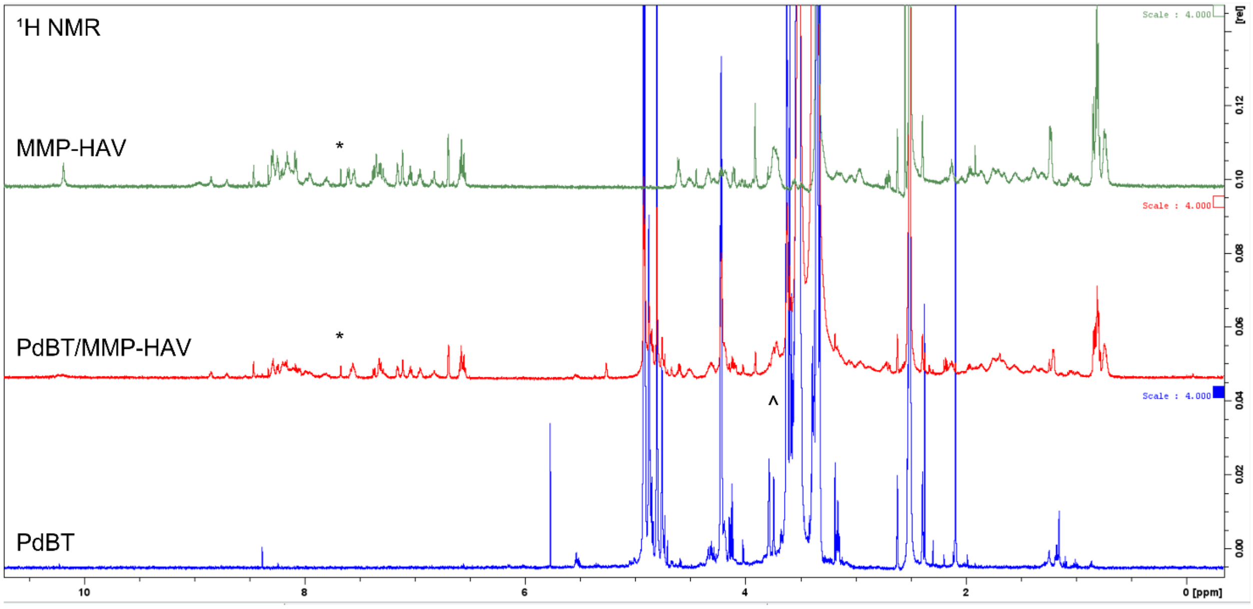

Supplementary Figure 6. [1] H NMR spectra of MMP-HAV, PdBT, and PdBT/MMP-HAV products dissolved in DMSO d6. The peak at 7.6 ppm (*) on the MMP-HAV and PdBT/MMP-HAV products correspond to the proton on histidine and the large peak at 3.54 ppm (^) on the PdBT and PdBT/MMP-HAV products correspond to the protons on the PEG backbone of PdBT.

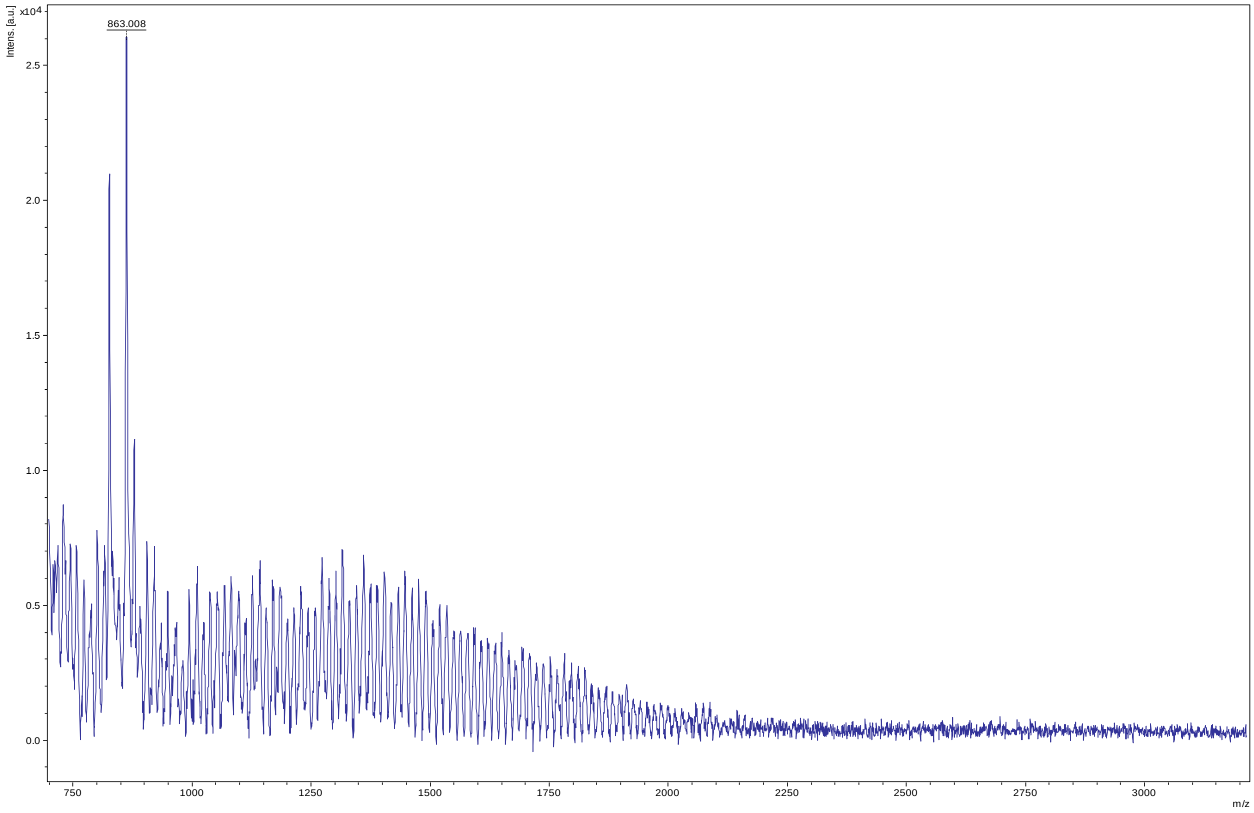

Supplementary Figure 7. MALDI-TOF spectrum for PdBT crosslinking macromer. The peak at 863 Da is PEG which is a comonomer for the creation of the product. The band observed between 1000 and 2000 Da represents the PdBT product.

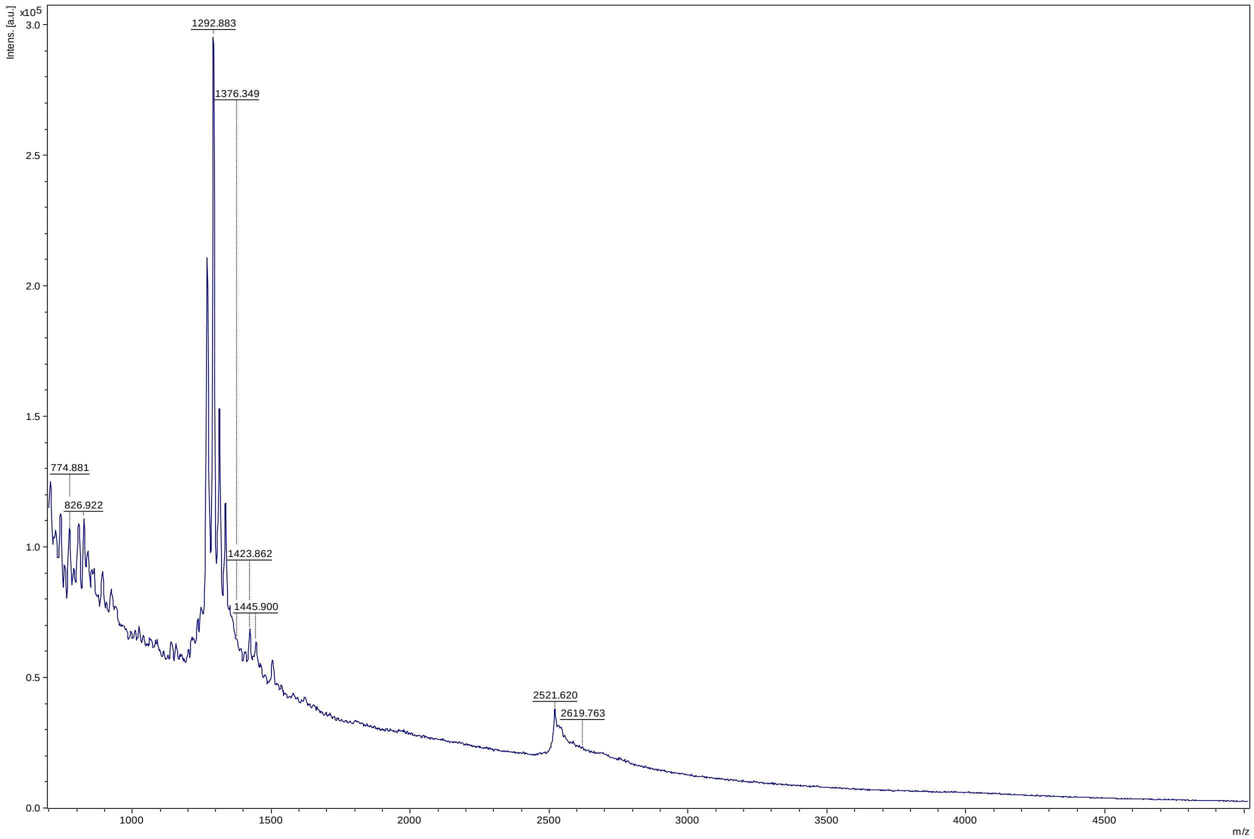

Supplementary Figure 8. MALDI-TOF spectrum for PdBT/HAV products. The peaks at 774 and 826 are PEG peaks as seen in the PdBT spectra above. 1292 Da is the HAV peptide. The PdBT/HAV product with one peptide conjugated per mol PdBT (2520 Da).

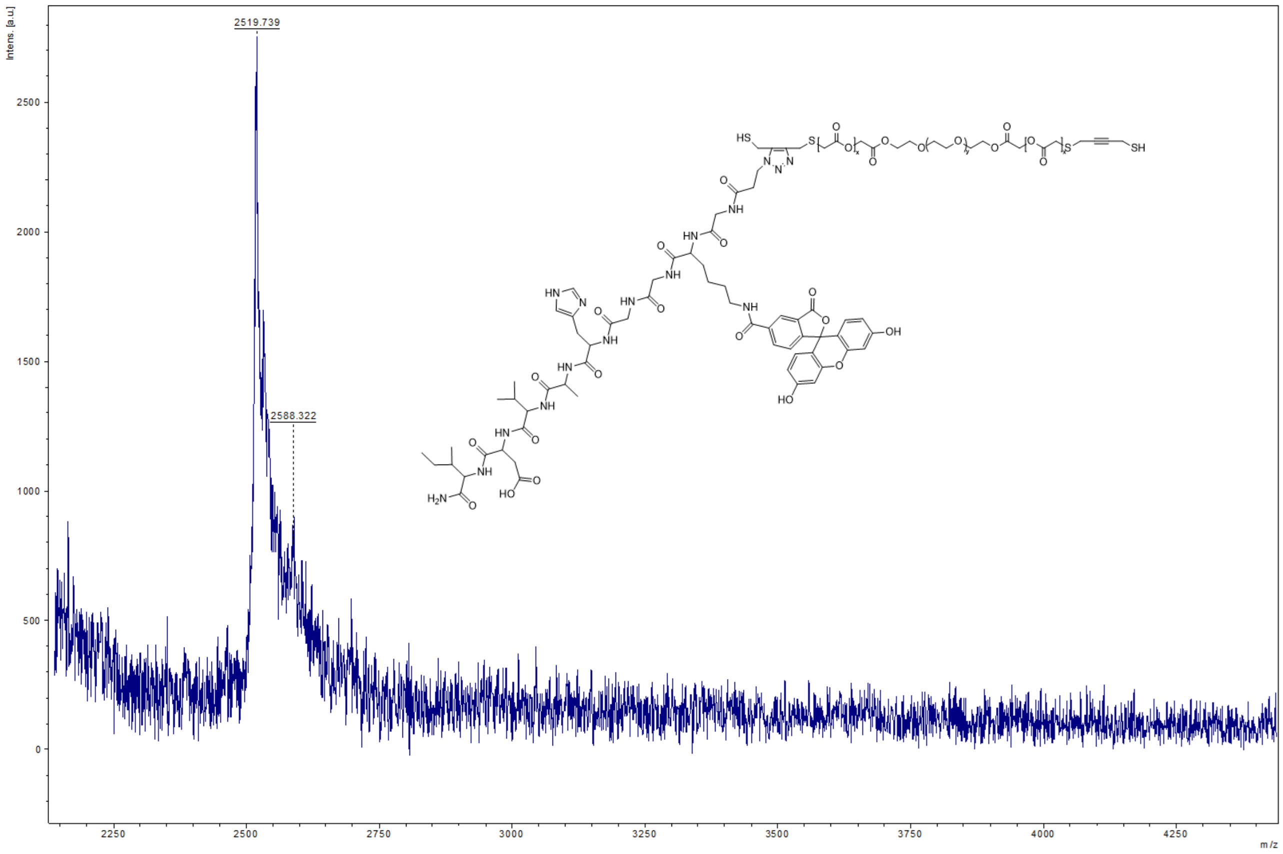

Supplementary Figure 9. Zoomed in view of the MALDI-TOF spectrum of PdBT/HAV conjugate.

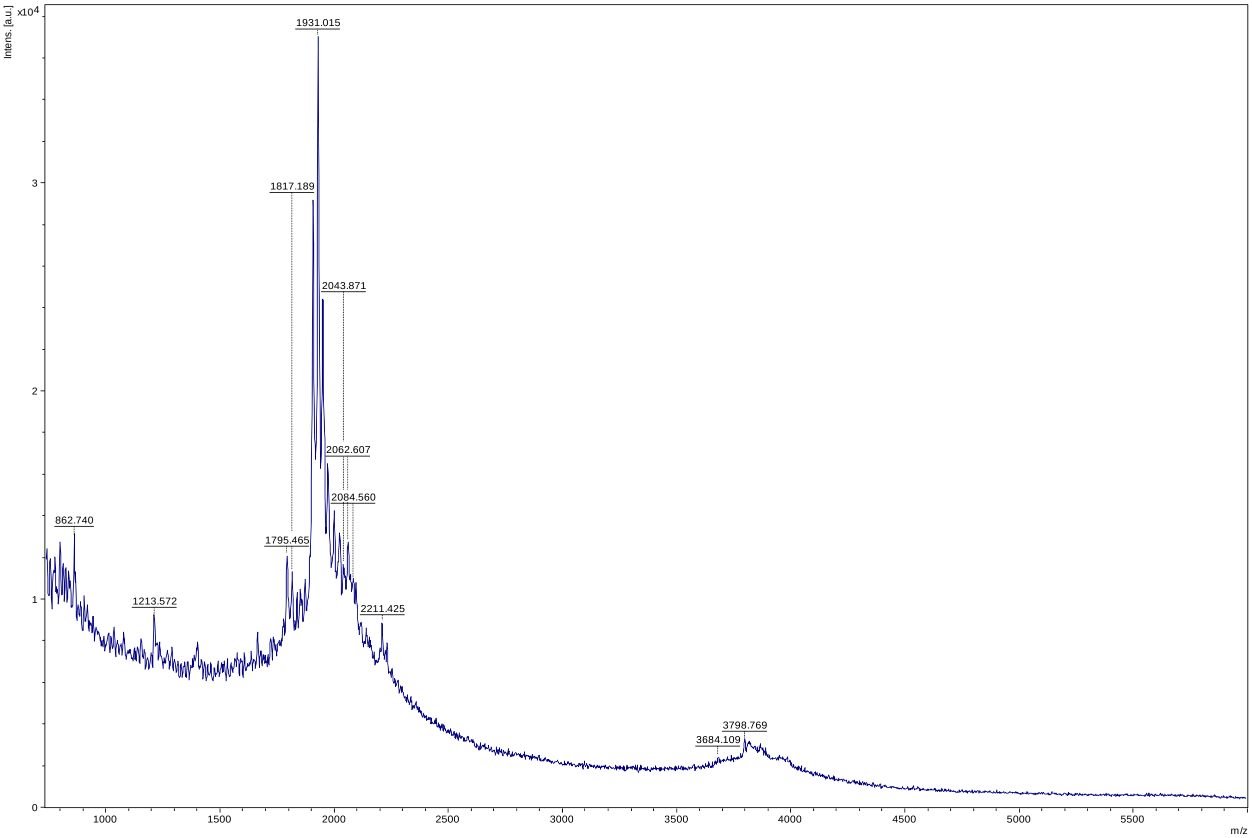

Supplementary Figure 10. MALDI-TOF spectrum for PdBT/MMP-HAV products. The peaks at 862 and 1213 are PEG as seen in the PdBT spectra above. The peaks between 1931 Da and 2211 Da are the MMP-HAV products plus NaCl, TFA, H_2O, etc. The peaks at 3800 Da are the PdBT/MMP-HAV product.

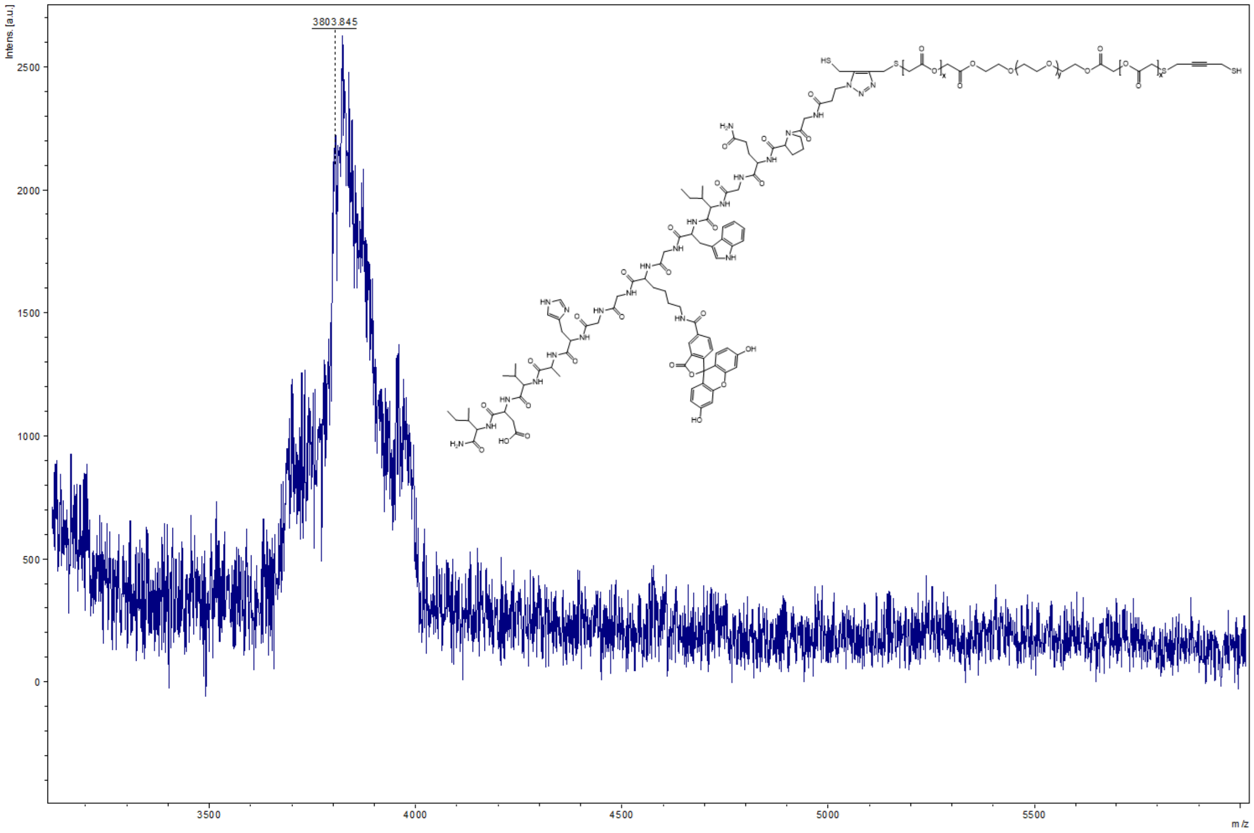

Supplementary Figure 11. Zoomed in view of the MALDI-TOF spectrum of PdBT/MMP-HAV conjugate.

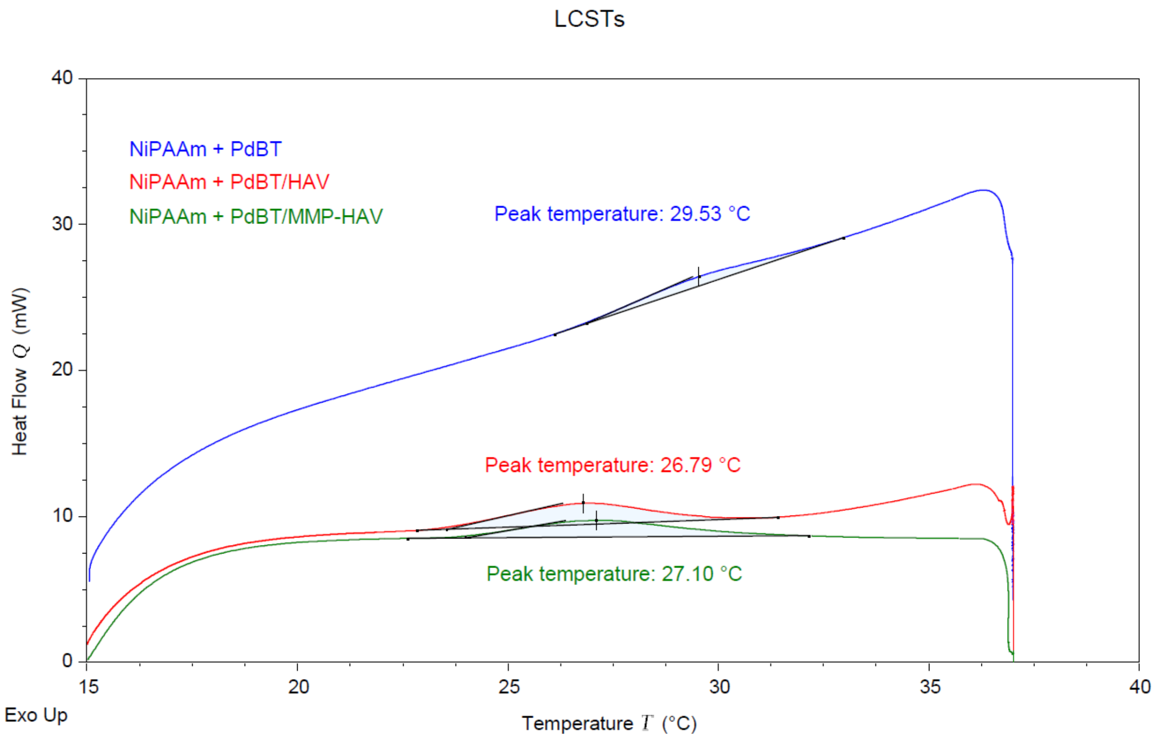

Supplementary Figure 12. LCSTs of hydrogel precursor solutions measured via DSC.

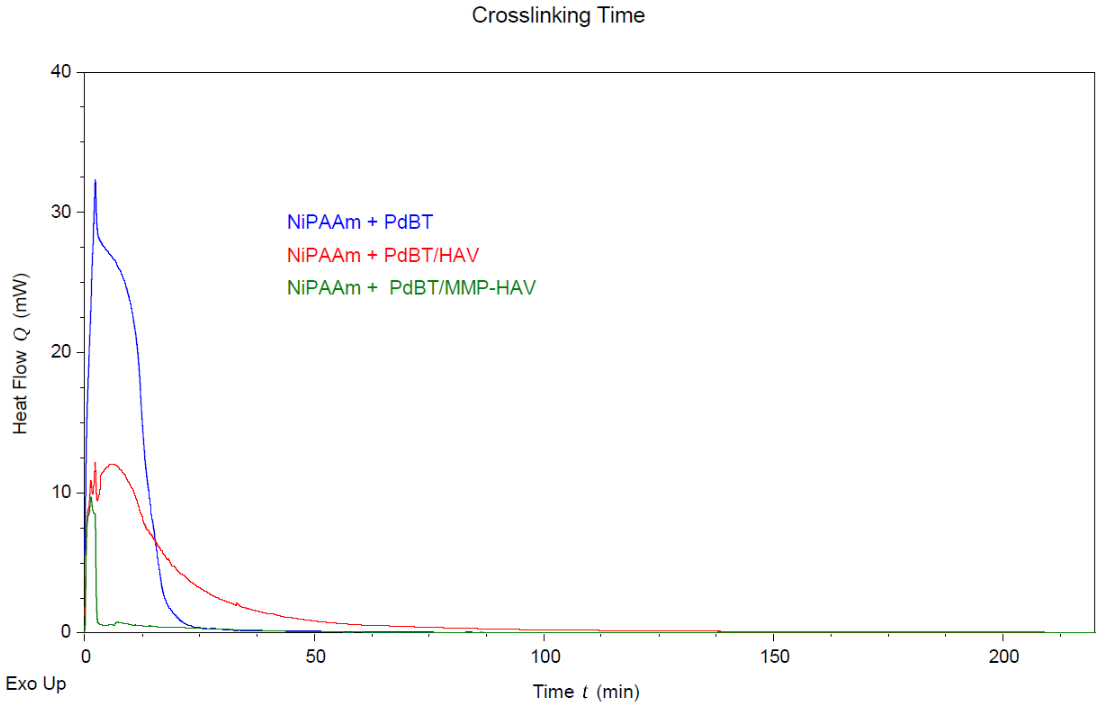

Supplementary Figure 13. Crosslinking time of hydrogels as measured via DSC.

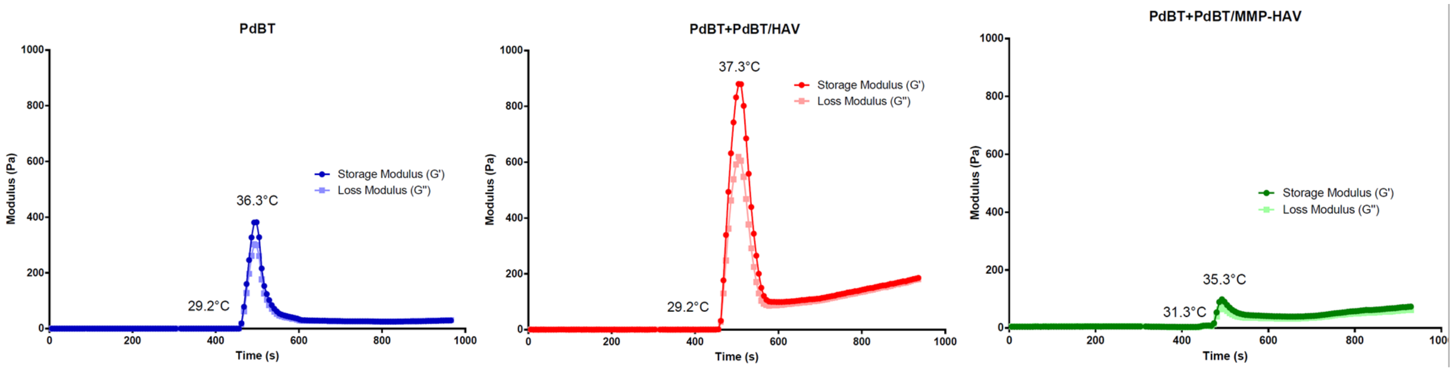

Supplementary Figure 14. Rheometry of gel compositions indicating onset and peak LSCTs demonstrating functional gelation.

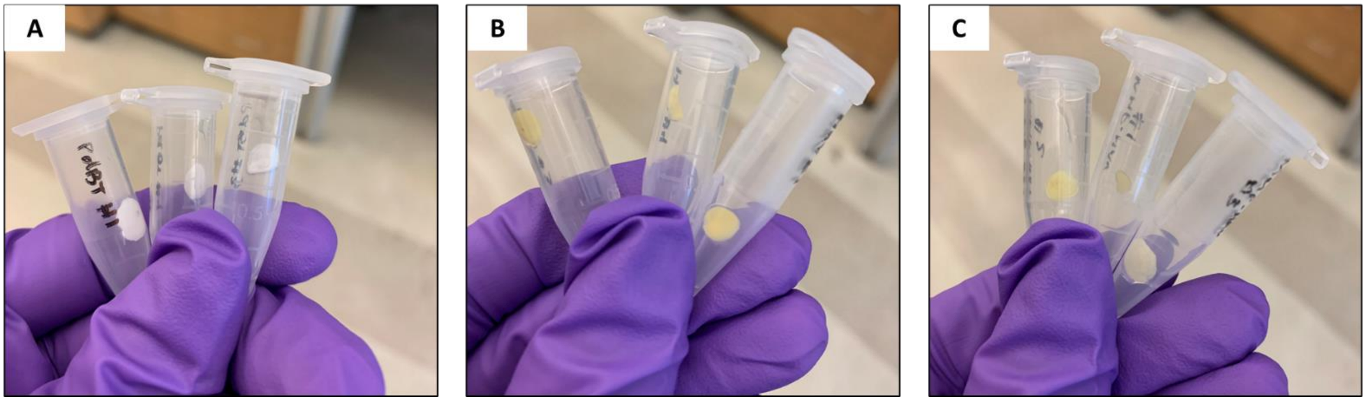

Supplementary Figure 15. Representative images of the **(A)** PdBT, **(B)** PdBT/HAV, and **(C)** PdBT/MMP-HAV hydrogels.

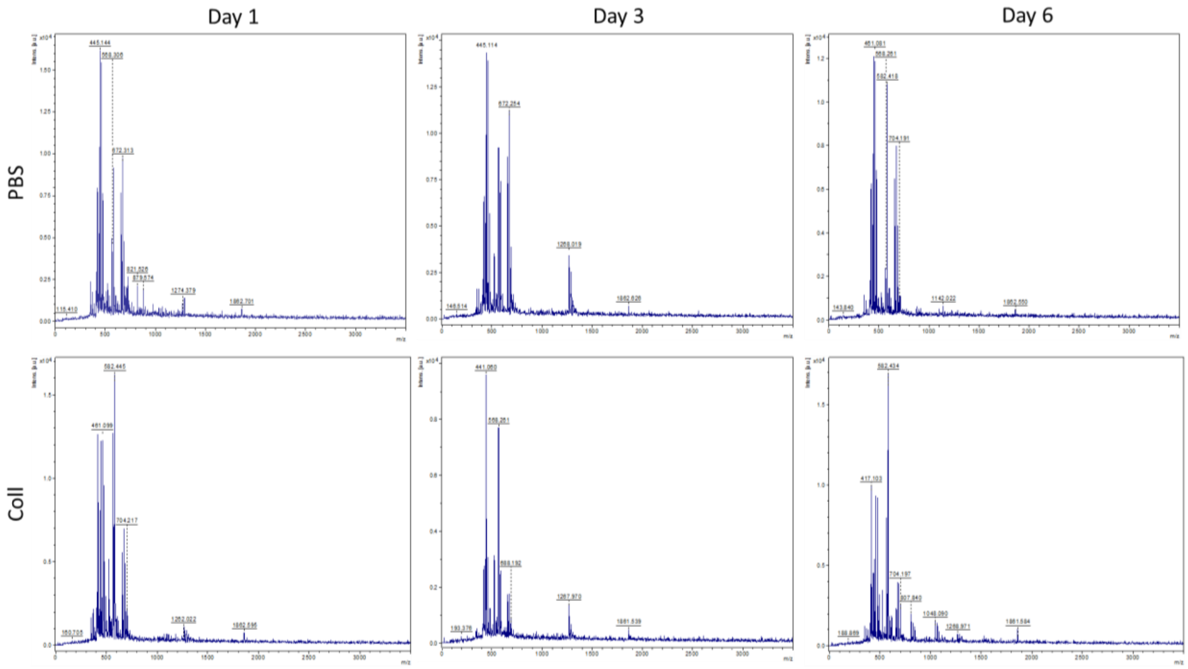

Supplementary Figure 16. MALDI-TOF spectra of degradation/release products for PdBT/HAV gels on days 1, 3, and 6. The peaks observed between 400-800 in all conditions represent varied HAV and PdBT degradation fragments. The peaks observed near 1280 Da represent a HAV peptide fragment released from the network. The peaks observed at 1860 Da is a PdBT/HAV degradation fragment.

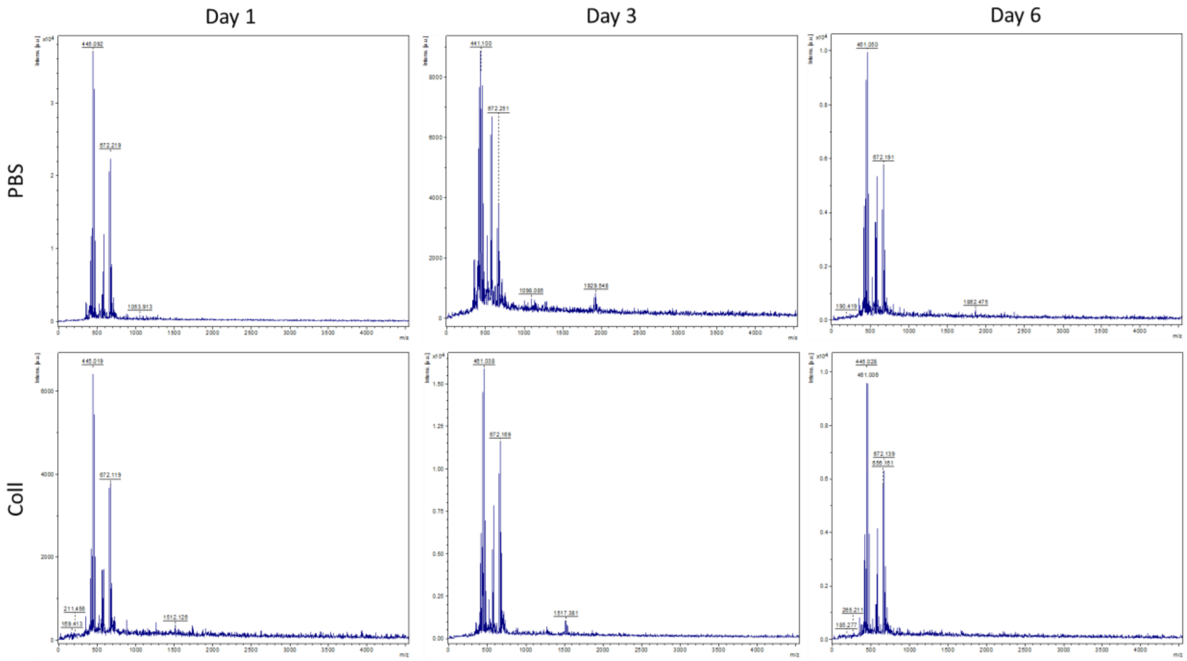

Supplementary Figure 17. MALDI-TOF spectra of degradation/release products for PdBT/MMP-HAV gels on days 1, 3, and 6. The peaks observed between 400-700 in all conditions, and 1050, 1100, 1930, and 1860 Da in PBS conditions represent varied MMP-HAV and PdBT degradation fragments. The 1512 and 1517 Da peaks observed in collagenase containing conditions for days 1 and 3 represent the sequence cleaved by the collagenase (WGKGGHAVDI + Fluorophore).
